# Measuring Violence Against Children: A COSMIN Systematic Review of the Psychometric Properties of Child and Adolescent Self-Report Measures

**DOI:** 10.1177/15248380221082152

**Published:** 2022-04-21

**Authors:** Franziska Meinck, Lakshmi Neelakantan, Bridget Steele, Janina Jochim, Lynn M. Davies, Mark Boyes, Jane Barlow, Michael Dunne

**Affiliations:** 1 School of Social and Political Science, 151027 University of Edinburgh, Edinburgh, UK; 2 OPTENTIA, Faculty of Health Sciences, 12205 North-West University, Vanderbijlpark, South Africa; 3 School of Public Health, 98584 University of the Witwatersrand, Johannesburg, South Africa; 4 Moray House School of Education, 1649 University of Edinburgh, Edinburgh, UK; 5 Department of Psychiatry, 1969 University of Oxford, Oxford, UK; 6 Department of Social Policy and Intervention, University of Oxford, Oxford, UK; 7 Curtin enAble Institute and School of Population Health, Faculty of Health Sciences, 1649 Curtin University, Perth, Australia; 8 Australian Centre for Health Law Research, 1969 Queensland University of Technology, Brisbane, Australia; 9 Institute for Community Health Research, Hue University, Vietnam

**Keywords:** violence against children, child abuse, child maltreatment, measurement, psychometrics, instrument

## Abstract

Research on violence against children (VAC) requires meaningful, valid, and reliable self-report by children. Many instruments have been used globally and decisions to select suitable measures are complex. This review identifies child and adolescent self-report measures that are most likely to yield valid, reliable, and comparable data in this field. A systematic review (PROSPERO: CRD4201706) was conducted using the 2018 Consensus-based Standards for the selection of health Measurement Instrument (COSMIN) criteria. Six electronic databases and gray literature were searched. Manuscripts published in English and describing the development and psychometric qualities of child/adolescent self-report instruments were included. Thirty-nine original instruments and 13 adaptations were identified in 124 studies. The quality of evidence ranged from “very low” to “high” depending on the measure and the psychometric properties assessed. Most measures were not widely used, and some have been applied in many settings despite limited evidence of their psychometric rigor. Few studies assessed content validity, particularly with children. The ACE, CTQ, CTS-PC, CECA, ICAST, and JVQ have the best psychometric properties. An overview of items measuring frequency, onset, duration, perpetrators, and locations is provided as well as an assessment of the practicalities for administration to help researchers select the instrument best suited for their research questions. This comprehensive review shows the strengths and weaknesses of VAC research instruments. Six measures that have sufficient psychometric properties are recommended for use in research, with the caveat that extensive piloting is carried out to ensure sufficient content validity for the local context and population.

## Background

In the past two decades, in many countries, there has been major social change underway as children, parents, educators, health workers, social care professionals, and political leaders call for action to prevent violence against children (VAC). Targets have been set within the 2030 Sustainable Development Goals for all countries to reduce violence in families and communities. This has created a need for solid evidence on the prevalence, causes, and consequences of childhood violence and many thousands of research studies have been published.

Arguably, however, the expansion of VAC advocacy and research has not been matched by gains in the quality of metrics used in many studies. Progress in the field has been hampered by the use of unstandardized measurements (Finkelhor et al., 2013; Moore et al., 2015), which has contributed to implausibly wide variation in estimates within and between studies (Fang et al., 2015). When statistical confidence is low, when findings appear not to be replicable, for example, when published prevalence estimates for emotional abuse range from 3% to 80%, and from 0.3% to 44% for child sexual abuse (Laurin et al., 2018), people who need to use research for social good may be confused about its meaning.

Reliable and valid measures are needed to improve understanding of trends in the occurrence of VAC and to determine whether prevention efforts are effective (Meinck et al., 2016). Good measurement enables robust estimations of the impact of modifiable risk factors for violence, and accurate assessment of change in preventive intervention studies. However, the sheer number of child abuse measures available can be daunting for those wishing to identify a rigorous measure for their research.

Selection of a self-report measure is also complicated by the fact that measures often have multiple versions. These generally include one or more of the following: (1) child self-report measures, generally used with children aged 12 and above, (2) retrospective self-report measures, used with adults and youth aged 18 and above, (3) parental proxy-report measures asking parents to report on abuse their children may have experienced, and (4) parental self-report measures assessing parent’s use of harsh discipline and corporal punishment. Among self-report measures, those using child self-report and adult retrospective self-report are most commonly used in surveys.

Five reviews have previously evaluated VAC child self-report measures (Mathews et al., 2020; Ritacco & Suffla, 2012; Saini et al., 2019; Tonmyr et al., 2011; Walsh et al., 2004) . Walsh et al. (2004) provide an overview of child sexual abuse measures, their psychometric properties and how these measures can be used to collect data. Tonmyr et al. (2011) reviewed measures for emotional/psychological maltreatment and assessed their validity and reliability. Ritacco and Suffla (2012) evaluated the psychometric properties of measures to assess prevalence, incidence, and intervention effectiveness, as well as applicability of measures in South Africa. Mathews et al (2020) conducted a systematic review of measures of at least four forms of maltreatment used in national prevalence studies. While these reviews are very important to the field, they are limited in the fact that some were not systematic reviews (Ritacco & Suffla, 2012; Walsh et al., 2004), focused on only one form of abuse (Walsh et al., 2004), in this case sexual abuse, or only on multiple forms of abuse, or included measures only if they had been used in national prevalence studies (Mathews et al., 2020), and therefore may have missed tools that are successfully applied more broadly in clinical, education and social research into VAC. One review included child-self report among many other types of self- and proxy report (Saini et al., 2019) and two reviews  did not conduct quality appraisal in a formalized way (Ritacco & Suffla, 2012; Walsh et al., 2004). Two reviews on the psychometric properties of child abuse measures have been conducted using COSMIN criteria: one focused exclusively on parent report measures of abuse (Yoon et al., 2020); the other on all types of self-report (parent, child and adult retrospective) which came to the conclusion that no instrument is superior over others (Saini et al., 2019). 

**Figure 1.**
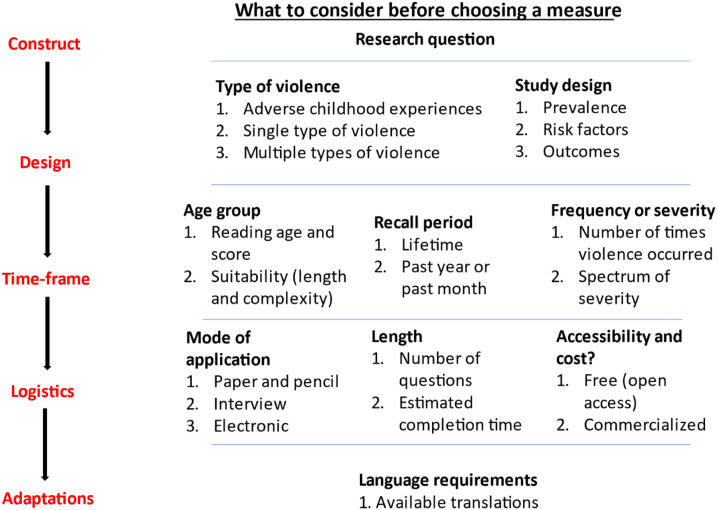
Considerations in measurement selection.

In addition to good psychometric properties, selection of an appropriate VAC measure is influenced by the specific research questions. Key features are summarized in Figure 1. These include the types of violence to be measured, the design of the study, the targeted age groups, the recall period, and whether one also wishes to establish frequency or severity or specific settings in which violence occurs. Consideration should also be given to the mode of application, number of items, language requirements and accessibility, and cost of the measures.

This systematic review therefore has four aims: (1) to identify all standardized current child and adolescent VAC self-report measures; (2) to describe the functionality, content, and structure of these measures in the contexts they were used; (3) to evaluate the psychometric properties of the identified measures; and (4) to provide evidence-based guidance in selecting an appropriate instrument for those wishing to conduct research on child abuse and neglect.

## Methods

This systematic review follows the Consensus-based Standards for the selection of health Measurement Instrument (COSMIN) Guideline for Systematic Reviews of Patient Reported Outcome Measures (Prinsen et al., 2018, Figure 2). The protocol for this review was registered on PROSPERO (2017:CRD4201706) on April 7, 2017.

**Figure 2.**
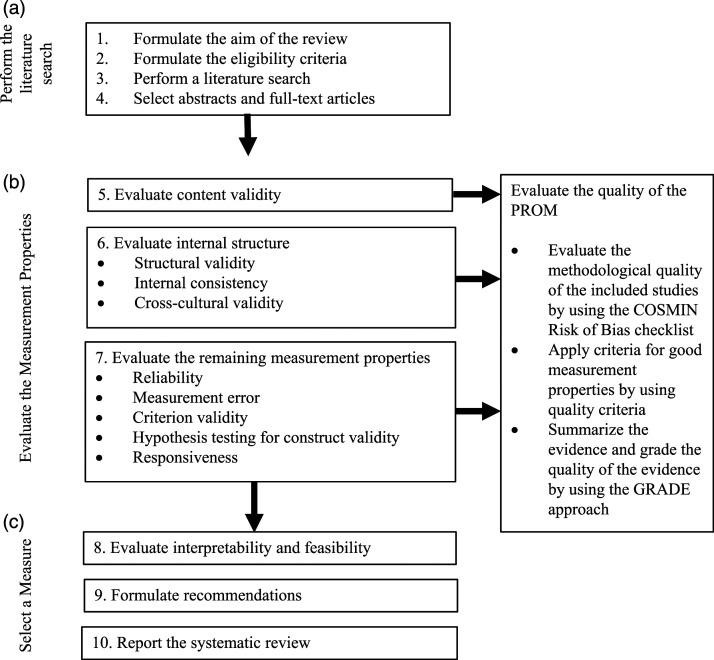
Ten steps for conducting a systematic review of PROMS (from: Mokkink, Prinsen, et al. 2018).

### Literature Search

The following databases were searched: PsycINFO, MEDLINE, Embase, Global Health, ProQuest, and Social Sciences Citation Index. The search strategy was piloted multiple times by one reviewer to increase specificity and sensitivity until an optimal set of final search terms was identified (Supplement 1). The searches spanned the time frame from the inception of the databases until April 30, 2017 and searched title, abstract, and keywords. Updated searches were completed up to October 12, 2020. Direct searches were carried out in the key academic journals reporting on empirical research into child abuse and neglect: Child Abuse Review, Child Abuse and Neglect, and Child Maltreatment. Further, abstract compilations of the international and regional conferences of the International Society for the Prevention of Child Abuse and Neglect (ISPCAN) and of the International Society for Child Indicators (ISCI) were directly searched.

In addition to the above, reference lists of retrieved articles were screened and relevant experts in the field were contacted to identify further studies, which may not have been identified through the searches. Further, forward citation searches were used with manuscripts that cited studies included in the review. Individual articles from other reviews were also retrieved.

### Selection Criteria for Eligible Studies

After the removal of duplicate studies, titles and abstracts were assessed. Studies were included if they met the following criteria: (1) the measure they reported on was designed to assess VAC (physical abuse; including harsh parenting, emotional abuse, sexual abuse, and domestic violence exposure and neglect) or assessed VAC as part of a sub-scale in a longer inventory; (2) the measure was used with children or adolescents; (3a) the study described the development or validation of a child abuse measure, or (3b) reported data on the content validity, structural validity, internal consistency, cross-cultural validity, reliability, measurement error, criterion validity, hypothesis testing for construct validity (with the following pre-specified outcomes which were associated with a history of childhood aversity with an odds ratio > 3.5 in a recent meta-analysis (Hughes et al., 2017): depression, anxiety, suicidal ideation, self-harm, problem behavior in the form of delinquency, criminal activity or violence perpetration and drug use or revictimization, different types of abuse measures, and other types of violence), responsiveness, or concordance of a measure; (4) used a validated outcome measure for the hypothesis testing for construct validity or a medical/clinical diagnosis; (5) the study was accessible in the English language; and (6) the study described the development or validation of a VAC measure using any of the psychometric properties mentioned above as part of an original analysis. Studies were excluded if they (1) did not measure VAC in the home/family environment but instead solely assessed another form of VAC, such as peer violence or community violence; (2) measured VAC by single items or without a standardized measure; (3) used any report other than child self-report; (4) conducted hypothesis testing for construct validity and did not include the pre-specified outcomes or otherwise measured pre-specified outcomes using a non-standardized measure; (5) measured attitudes and perceptions rather than abusive events; (6) cited psychometrics from a different study; (7) studies were not accessible; and (8) were published in languages other than English. When in doubt about the eligibility of study, the full text was retrieved and reviewed. Where there was still doubt after the full text was retrieved, the study was discussed by the author team. For all these cases, consensus was reached. The first author (FM) double-screened 5% of all identified articles during the screening stage and 10% of eligible articles. Each included article was verified for inclusion by a second reviewer prior to extraction.

Adapted versions of several the measures (i.e., to make it shorter or modified response options) were identified. These were included as independent measures.

### Data Extraction

The following data were extracted from each included publication: the measure, the intended construct for measurement, the administration method, the study population, the number of participants, the participant demographics, the country and setting, and the language. Specific details were also extracted from each study based on the psychometric property investigated, after which, quality assessment of the included studies was conducted for each study. Further information was extracted on presence or absence of additional items around perpetrators, locations, frequency, and severity of abuse.

### Methodological and Measurement Quality Assessment

The COSMIN guidelines were used for this systematic review (Mokkink, Prinsen, et al., 2018). These followed four steps to methodological and quality assessment. We evaluated, where possible, the following: First, the methodological quality of measure development and content validity studies (Terwee et al., 2018) and methodological quality of studies conducted on the psychometric properties of each instrument using the COSMIN Risk of Bias Checklist (Mokkink, Prinsen, et al., 2018). Second, we evaluated the quality of content validation procedures for each included study and each of the measure’s psychometric properties as they were presented in each study (Prinsen et al., 2018). These are organized using COSMIN criteria that relate to internal structure (structural validity, internal consistency, and cross-cultural validity) and other measurement properties (reliability, measurement error, criterion validity, hypothesis testing for construct validity, and responsiveness. We also added concordance). Third, we applied the modified Grading of Recommendations Assessment, Development and Evaluation (GRADE) to examine the quality of the overall body of evidence for each instrument (Prinsen et al., 2018). All COSMIN guidance is available at www.cosmin.nl. Fourth: for each instrument, we assessed practical administrative properties.

### Step 1: Assessment of Methodological Quality of Included Studies

The methodological quality of included studies was assessed using the COSMIN Risk of Bias checklist (Mokkink et al., 2018). For content validity studies focused either on measurement development or additional content validity studies, the assessment of content validity requires the assessment of the quality and results of the individual study as well as the content of the measure itself. All content validity studies were assessed in terms of relevance, comprehensiveness, and comprehensibility (Terwee et al., 2018). Relevance requires that all items in a measure should be relevant for the construct of interest in a specific context and with a specific population. Comprehensiveness requires that no key aspects of the construct should be missing, and comprehensibility demands that the items and their response options should be understandable by the target population as intended.

For studies of other measurement properties, assessment focused on appropriateness of the study design, methodology, and the statistical analyses employed.

Each measurement property was rated across several items assessing the methodological quality of the study. The four response options used were very good, adequate, doubtful, and inadequate. The overall score of the methodological quality of the study was determined by the lowest rating across the checklist, for example, a study may have scored very good on the design requirement items but inadequate on the statistical methods and would then receive an overall score of inadequate. Where insufficient information was provided to assess the quality of the study, an inadequate rating was given in line with COSMIN guidance (Mokkink, Prinsen, et al., 2018; Prinsen et al., 2018).

### Step 2: Assessment of Study Results for Each Psychometric Property

All study results for each included study and measure were assessed according to the COSMIN guidance on good measurement properties (Prinsen et al., 2018). Content validity was evaluated based on the content validity of the measure itself and the quality of the available studies (Terwee et al., 2018). Content validity was scored as sufficient (+), insufficient (−), indeterminate (?), and inconsistent (±) based on existing development studies, content validity studies, and reviewer ratings (Supplement assessment form 1). Where no content validity studies were available, no rating was given. When an article described the translation of an instrument and none of the the reviewers spoke the language of the translated instrument, an indeterminate (?) rating was given for content validity reviewer scoring. All studies, even those whose quality was judged “inadequate,” were rated in order to gain a comprehensive overview of the included outcome measures.

The COSMIN guidance checklist was used for the following 10 psychometric properties: structural validity, internal consistency, cross-cultural validity, reliability, measurement error, criterion validity, hypothesis testing for construct validity, responsiveness, and concordance of a measure. These fall into the measurement domains of validity (construct validity: structural validity, cross-cultural validity, hypothesis testing, concordance, and criterion validity), reliability (test–retest reliability, internal consistency, and measurement error), and responsiveness (Prinsen et al., 2018). For each of the outcomes, the rater was required to assign a rating of sufficient (+), insufficient (−), indeterminate (?), or inconsistent (±). Studies were considered sufficient if they used appropriate statistical procedures and demonstrated appropriately high scores (Supplement assessment form 2). Indeterminate ratings were given when information was not fully reported, or hypotheses were missing. An inconsistent rating was given in cases when some hypotheses were met but others were not. For hypothesis testing for construct validity, the decision was made to only include the following outcomes: other VAC measures as a comparator, or any of the following pre-specified outcomes that are associated with VAC and related adversity with odds ratios > 3.5 in a recent meta-analysis (Hughes et al., 2017): depression, anxiety, suicidal ideation, self-harm, problem behavior in the form of delinquency, criminal activity or violence perpetration or revictimization, and drug use. For criterion validity, as there is no gold-standard measure for VAC, only studies that reported correlations between a VAC measure and substantiated cases of child abuse were included. We also included an additional psychometric category, concurrence, which was used for studies that compared two different reports of the same act (e.g., child and parent report). This would not qualify for inter-rater reliability as the questions were often different. For example, parents could be asked about their use of disciplining techniques with regards to their child while children could be asked about all the forms of violence they experienced at the hands of their parents and others. Studies assessing specificity and sensitivity of the measures were also eligible for inclusion.

### Step 3: Summary and Quality Grading of the Evidence

The psychometric findings reported in each of the studies and for each of the measures were summarized and graded according to COSMIN criteria (Prinsen et al., 2018). Measures were assigned two overall ratings. First, methodological quality was assessed for each of the measurement properties and the evidence was graded as “high,” “moderate,” “low,” or “very low.” This grading considers multiple factors including inconsistency of results, imprecision through small sample sizes, and evidence for risk of bias, as well as the number of available studies and the methodological quality of each study. Second, each of the psychometric properties (except content validity) were given sufficient (+), insufficient (−), indeterminate (?), or inconsistent (±) ratings. Where the rating was inconsistent for a measure on one of the psychometric properties, the quality of the body of evidence was not graded in line with COSMIN criteria.

### Step 4: Assessment of Practical Administrative Properties

Following the quality assessment, measures were assessed for their applicability in research. Time for administration, mode of administration, ease of scoring (availability of a handbook), readability and comprehension, availability (ease of accessibility, open source, or user-pays), conditions of use (qualifications of interviewers/technical requirements), and published translation into multiple languages were assessed. In addition, we assessed whether the instrument included questions on perpetrators, settings of abuse, disclosure of abuse, severity, and burden of participation. We also recorded whether the instrument included a validity/lie scale.

## Results

### Description of Studies

**Figure 3.**
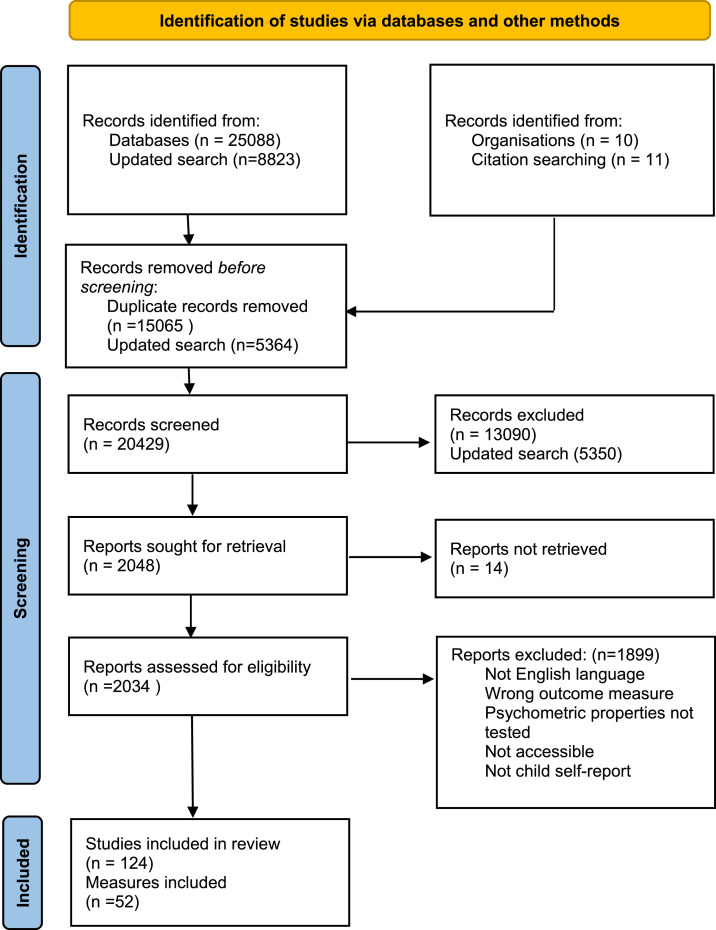
PRISMA flow diagram (from: Page et al. 2021).

The original search identified 33,911 articles. After removing duplicates, 20,429 articles were identified for screening. After screening, 2034 full text articles were retrieved and assessed for eligibility. An additional 11 studies were found in previous systematic reviews. Of all assessed studies, 124 met the inclusion criteria for this review. 52 VAC child self-report measures were identified, of these 39 were original measures and 13 modified versions (including other language versions) of the original studies. The study selection flow chart is shown in Figure 3. Included studies can be found in Supplement 2.

Study publication dates ranged from 1989 to 2020. Diversity of the study populations within studies was not particularly highlighted. Studies included in the review were conducted in countries spanning most world regions including Western, Central, and Eastern Europe, sub-Saharan Africa, North and South America, the Middle East, Southeast and East Asia, and as such across the review contained diverse populations. Study sample sizes ranged from 58 to 42,194, including nationally representative samples, clinical populations, male and female only samples, and samples of ethnic minorities. The majority of studies reported hypothesis testing for construct validity, whereas 10 studies focused on measurement development or content validity and 39 were instrument validation studies.

### Description of Measures

A description of each of the measures, original and modified versions is presented in Supplement 3 and 4. Forty measures assess multiple forms of VAC including physical, emotional, and sexual abuse, neglect and exposure to domestic violence. Thirteen measures assess a single form of violence (five physical violence, one psychological violence, five sexual violence, and two neglect). Eight measures focused on exposure to childhood trauma including physical, emotional, or sexual abuse and neglect. Seven child self-report measures focused on aspects of parenting and included sub-scales for corporal punishment, physical discipline, or harsh parenting. All measures included in this review ask about specific actions that constitute VAC rather than whether or not a participant felt they were being abused. Questionnaires either use screeners that score whether a specific type of violence was experienced and then participants are asked follow-up questions (if affirmative), or the instruments give participants an immediate option of reporting on the frequency in which a specific type of violence has occurred (e.g., “never,” “once or twice,” “at least once a month,” “weekly or more often,” “never,” “sometimes,” and “a lot”).

The vast majority of measures are targeted at children and adolescents aged 10 and above. Seven measures were specifically developed for younger children starting from age 5 (Bhat et al., 2012; Essau et al., 2006; Hecker et al., 2016; Malik & Shah, 2007; Nordstrom-Klee, 2001; O’Boyle, 2002; Sierau et al., 2018).

The majority of measures were developed in high-income countries (HIC), mostly North America or by researchers based at universities in HIC (*n* = 49). Despite this, the vast majority of instruments were designed for use across languages and contexts. For inclusion, all papers had to be written in English, but the measures did not have to be in English if the paper was written in English. Measures were available in various languages including English, German, Spanish, Arabic, Turkish, Portuguese, Croatian, Mandarin, Farsi, Malay, Hebrew, Swedish, Persian, Swahili, isiXhosa, chiChewa, Romanian, Urdu, Albanian, Bosnian, Greek, and Serbian (Supplement 4).

### Measurement Properties

One-hundred and twenty-four studies reported on at least one psychometric property of 52 measures (Supplement 3). Thirty-nine were original measures and 13 modified versions of these original measures. The largest number of modified versions was available for the ICAST, the ACE, and the CTQ.

The majority of studies reported on hypothesis testing for construct validity, followed by a smaller number of studies that reported internal consistency, factor structure, or reliability. Only 10 studies reported on content validity. No studies reported on the measurement error of the included measures. Similarly, no studies reported on the sensitivity or specificity of VAC measures, which we had set out to assess in addition to COSMIN criteria.

### Content Validity Assessment

Ten out of 52 measures described an aspect of content validity. Seven original measures and three modified versions were assessed. As described above, relevance, comprehensiveness, and comprehensibility of the four original measures and two modified versions were rated separately in a multi-step process in line with COSMIN guidelines (Terwee et al., 2018).

First, the quality of the development of the five measures that reported on development were evaluated (Supplement 5). One original measure was rated inadequate (ICAST-CH), one doubtful (CAS1), one adequate (CASRS), and two very good (CECA.Q and CTS). One modified measure was rated inadequate (ICAST-CI). A total of five out of 52 studies evaluated content validity either among the target participant group (children) or among professionals (e.g., psychologists and researchers). Relevance was investigated in only four studies with child participants and four studies with professionals. Comprehensiveness was assessed in two studies with children and in three studies with professionals. Comprehensibility was assessed in six studies with children. Children were, for the most part, not involved in the development and testing of measures for VAC and only one study specifically recruited children that may have had a history of abuse (Hamby et al., 2000). From all the studies assessing relevance among children and/or professionals, only one study received a “very good” rating (Meinck et al., 2018), two an “adequate” rating (Hamby et al., 2000; Mohammadkhani et al., 2003), and two a “doubtful” rating (Malik & Shah, 2007; Walsh et al., 2008). Of the few studies assessing comprehensiveness among children and/or professionals, one study received an adequate rating (Hamby et al., 2000), and two studies received a doubtful rating (Malik & Shah, 2007; Walsh et al., 2008). Of the few studies investigating comprehensibility among children, two were rated as very good (Meinck et al., 2018; Shirinbayan et al., 2020), one as adequate (Hamby et al., 2000), and two as doubtful (Malik & Shah, 2007; Silveira & Grassi-Oliveira, 2016; Walsh et al., 2008).

The quality of content validity for each included study was also assessed (Supplement 6).

#### Relevance

For development studies, the CAS1, CASRS, and CTS were assessed as having sufficient quality of content validity in relation to relevance. For content validity studies, the ICAST-Trial C and the JVQ were assessed as having sufficient content validity in relation to relevance. The reviewers assessed all measures as having sufficient relevance.

#### Comprehensiveness

In terms of comprehensiveness, no development study was rated sufficient. However, some content validity studies on the CAS1, the CEVQ, and the JVQ were rated as sufficient and the reviewer ratings for the CAS1, CEVQ, CECA, ICAST-CH, ICAST-CI, ICAST-Trial C, and JVQ were also sufficient.

#### Comprehensibility

In development studies, only on study on the CTS received a sufficient rating for comprehensibility. In content validity studies, only the studies on the ICAST-CH and the JVQ were rated sufficient for comprehensibility. The reviewer rated all measures in English language as sufficient forcomprehensibility.

Then the quality of the content validity for each outcome measure was evaluated (Supplement 7). The CEVQ, CECA, CASRS, CTS, and JVQ were rated sufficient for relevance, while the CAS1, the ICAST-CH, ICAST-CI, and ICAST-Trial C received an inconsistent rating. The CAS1, CEVQ, CECA, and JVQ were rated sufficient for comprehensiveness while the ICAST-CH and ICAST-Trial C received an inconsistent rating and the CARS, CTS, and ICAST-CI could not be rated for this domain. The CAS1, CECA, CTS, ICAST-Trial C, and JVQ were rated sufficient for their comprehensibility, while the CEVQ and ICAST-CH were rated as inconsistent and the CASRS and ICAST-CI could not be rated as studies that did not report on comprehensiveness or comprehensibility were not rated for these domains.

### Construct Validity

#### Structural validity testing

Structural validity was tested in 26 studies (Supplement 8 and 9). The methodological quality of studies testing structural validity ranged from very good to inadequate (Supplement 8). The quality of structural validity for studies that conducted principal component analysis or exploratory factor analysis without presenting appropriate fit indices was rated “insufficient” in line with COSMIN guidelines (Supplement 9). The ACE-ASF, ACE-IQ, CASRS, CECA, ICAST-Trial C, JVQ, MPQ, PPS, MNBS-CR, and SPaD received a “sufficient” rating for quality of structural validity (Supplement 10). All other measures either received an “insufficient,” “indeterminate,” or “inconsistent” rating. The quality of the body of evidence for instruments with “sufficient” structural validity ranged from moderate to high.

#### Cross-cultural validity/measurement invariance

Only one study could be rated for cross-cultural validity even though multiple studies adapted measures to different cultural contexts, ethnic groups, and languages, or used measures across multiple contexts and compared estimated prevalence. Many studies included back-translation or consensus-based group translations. The ICAST-C was rated “sufficient” with “high” quality of evidence based on one study.

Two studies for two measures could be rated for measurement invariance across sex (males/females). The APQ received an “insufficient” rating with moderate quality of evidence, the ACE-SF received a “sufficient” rating with a “very low” quality of evidence (Supplement 10).

#### Criterion validity

Only one study assessed criterion validity by comparing a modified version of the ICAST, the SC-ICAST-C, to the original ICAST measure. The study was rated as very good and the criterion validity as sufficient which resulted in an overall “sufficient” rating with a body of evidence rated as “high” for the SC-ICAST-C (Supplement 10).

#### Hypothesis testing for construct validity

One hundred and seven studies conducted hypothesis testing with information about this psychometric property available for forty-two measures (Supplement 8 and 9). Study quality was rated as ranging from very good to inadequate (Supplement 8). The quality of the psychometric dimension hypothesis testing ranged from sufficient to insufficient (Supplement 9). The quality of hypothesis testing was rated “sufficient” for ACE, APQ, CARI, CAS2, CASRS, CEVQ, CMQ, CTI2, CTQ, CTQ-SF, CTS-PC, CTS-PC-P, CTS, GPBS, ICAST-CH, ICAST-CI, ICAST-Trial C, JVQ, Kid-SAVE, LITE, M-ACE, MPQ, pediMACE, PPS, SC-ICAST-CH, TEQ, TESI, TISH, VHQ, CSAQ2, SAEQ, SES, CAPM, CMH-SR, CNQ, MNBS-CR, SAIPVEC, SPaD, and SRF (Supplement 10). The quality of the body of evidence conducting hypothesis testing ranged from very low to high, with most studies assessed as high quality.

#### Concordance

Sixteen studies had available data on concordance between child and parent report (Supplement 8 and 9). The quality of the studies ranged from very good to inadequate (Supplement 8) and concordance was rated from “insufficient” to “sufficient” (Supplement 9). The parent-child concordance for CTQ, JVW, SES, and MNBS-CR was rated “sufficient.” The quality of the evidence-base was high, except for the MNBS-CR which was rated moderate (Supplement 10).

### Reliability

#### Test–retest reliability

Twenty-two studies investigated test–retest reliability for 15 measures. The methodological quality of studies testing internal consistency ranged from very good to inadequate (Supplement 8). The quality of reliability within these studies was rated from insufficient to sufficient (Supplement 9). The CASRS, CECA, CEVQ, PPI2, TISH, MNBS-CR, and SPaD were rated “sufficient” (Supplement 10). All other measures either received an “insufficient,” “indeterminate,” or “inconsistent” rating. The quality of the body of evidence investigating reliability ranged from very low to high.

#### Internal consistency

Internal consistency was assessed for 35 out of 52 measures based on 65 studies (Supplement 8 and 9). The methodological quality of studies testing internal consistency ranged from very good to inadequate (Supplement 8). The quality of internal consistency within these studies was rated from insufficient to sufficient (Supplement 9). The APQ, CASRS, CEVQ, CTS-PC-P, CTS, ETI-SF, ICAST-CI, MACE, PPPS, CAPM, CNQ, and FAST received a “sufficient” rating (Supplement 10). All other measures either received an “insufficient,” “indeterminate,” or “inconsistent” rating. The quality of the body of evidence for measures rated “sufficient” for internal consistency ranged from very low to high.

#### Measurement error

No studies investigated measurement error and it was thus omitted from the table.

### Responsiveness/Sensitivity to Detect Change

Six studies investigated the responsiveness of the measure to detect change for five measures (Supplement 8 and 9). Study quality ranged from very good to inadequate (Supplement 8). In four studies, the responsiveness of the measures was rated “sufficient,” in two as “insufficient” (Supplement 9). The APQ, ICAST-Trial C, and MICS received a “sufficient” rating with a high to moderate quality of evidence based on one study each. The CTS-PC Child Version received an “inconsistent” rating based on three studies. The quality of the evidence for the CTS-PC Child Version could not be graded because COSMIN recommends not to grade when the psychometrics evaluation is rated “inconsistent.”

### Assessment of Broader Attributes of the Instruments

In addition to psychometrics, many other qualities of instruments should be considered when selecting an appropriate measure for a study on VAC. The following summarizes items that do not feature on all instruments but may be important in the selection of measures for future research.

#### Assessment of abuse properties

In VAC research additional questions on perpetrators, frequency, disclosure etc., can be important and as such, this additional information was also extracted (Supplement 3).

#### Measurement of perpetrators

The ACE-IQ, ACE, CAS2, CMIS-SF, CMQ, CEVQ, CECA, CARI, CTI2, FAST, ICAST, JVQ, LONGSCAN, MACE, MNBS-CR, CTS-PC-P, CTS-PC, PPS, PPI2, TESI-C, C-SARS, CSAQ1, SAEQ, CAPM, APQ, GPBS, MPQ, MICS, SPaD, SAIPVEC, and VHQ asked respondents to indicate information about the perpetrator/perpetrators of each abusive behavior. Of these, the APQ, CAPM-CV, CTS-PC, FAST, GPBS, MPQ-A, MNBS, MICS, PPS, PPI2, and SPaD only measured perpetration by parents/primary/secondary caregivers, for example, how often has your mother hit, punched, or slapped you?

##### Measurement of settings in which VAC occurs

Only the ICAST-CI (now discontinued and absorbed in ICAST-C V3.0), the JVQ extended version, the SIFAR, and the CSAQ1 have items on setting in which the abuse occurred.

##### Measurement of violence disclosure and response to disclosure

Few measures included items on VAC disclosure and response to disclosure. These included the CEVQ, the CTI2 (sexual abuse items only), LONGSCAN, C-SARS, CSAQ1, CSAQ2, SAEQ, and the JVQ.

##### Measurement of the severity and frequency of abuse

Most measures included items that assessed frequency. These had a range of response options ranging from never to often, almost never to almost always, and never to more than once a week. The only measures that did not assess frequency were the CTS, ETISR-SF, LONGSCAN, SIFAR, C-SARS, CSAQ1, CSAQ2, SAEQ, MPQ, and SAIPVEC.

A few measures also assessed onset or duration of abuse. These included the CEVQ, CARI, CSAQ, CTI2, pediMace, JVQ, and LITE-S.

Severity was assessed in multiple ways. Some instruments created a severity score across multiple items (e.g., mild to severe experiences), some asked about the short-term and long-term impact of the abuse exposure on the child, and some about sustained injuries. The instruments assessing severity in one of these ways were CMIS-SF, CASRS, CAS2, CMQ, CTS, CEVQ, CECA, CTQ, CTQ-SF, CARI, CTI2, JVQ, KID-SAVE, LITE, pediMACE, MACE, CTS-PC, CTS-PC Picture, SIFAR, TEQ, TESI-C, and SAEQ.

##### Validity scale/minimization-denial scales

Validity sub-scales or minimization-denial scales are used to determine a response bias in retrospective reports of childhood trauma that minimizes the extent of the trauma experienced. Items on such a scale might state, for example, “my childhood was fantastic.” Of all measures included in this review, only the CTQ and CTQ-SF had a minimization-denial scale.

##### Assessing the burden of participation (Supplement 4)

Only three measures included items on participant burden which assessed how difficult participants found the questions, if they got upset and if they would participate again: the ICAST, CEVQ, and JVQ.

#### Assessment of practical administrative properties (Supplement 4) Accessibility, administration time, ease of scoring, readability and availability in multiple languages was also assessed.**

##### Accessibility (Supplement 4)

Many of the measures were free to access, easy to find online and free to use for researchers. The ACE, JVQ, APQ, CEVQ, CECA, MICS, and LONGSCAN measures seem to be used very frequently in international research and have their manuals and procedures available online. The ICAST is free to use but must be requested from ISPCAN. Several measures were available within appendices of dissertations or publications, with some requiring subscription to research databases. Most of the papers did not indicate the measures’ copyright status. There were some measures which were not available to use for free for research purposes: the CTQ and CTS-PC, which are licensed and require payment for access, the manual and score sheets/application, and the ETISR-SF, which requires payment for application.

Notably, the CTQ and CTS-PC were the only measures that require certification by a professional organization for those applying the instrument, while the pediMACE and TESI-C require application by clinicians. Authors of all other questionnaires recommend administration or at least oversight by well-trained interviewers who have experience in working with vulnerable children and can enact distress protocols. The following measures were not retrievable for assessment by the research team: CMQ, CAS2, CAPM-CV, SAIPVEC, and SRF.

##### Time to administer (Supplement 4)

Most papers did not state the time typically needed for administration. For those that did, completion time ranged from 5 minutes for CTQ-SF, FAST, LITE-S, ACE-ASF, TEQ to more than 30 minutes for CECA.Q, SAEQ, and TESI. The other measures ranged between 10 and 30 minutes for completion. Depending on the complexity of the questions and response options, the number of questions approximately reflected the amount of time needed to complete the measure. Children without violence exposure complete measures much faster than children with violence exposure, particularly on measures that offer more complex response options than “yes” and “no.” This is because children with violence exposure need time to estimate frequency and perpetrators, age of onset, severity, etc., which their non-abused peers do not require.

##### Ease of scoring and availability of a technical manual or handbook (Supplement 4)

The following measures have a handbook that supports application and scoring: ACE, ICAST-CH, APQ, CTS, CTQ, CARI, Comprehensive Trauma Interview, CTSPC-R, CTS-PC, CTS-PC Picture, ETISR-SF, LITE-S, pediMACE (part of a dissertation), MACE, LONGSCAN, CECA.Q, SAEQ, JVQ, SES, TESI-C, TEQ, MNBS, MICS, TISH, SIFAR, and VHQ.

Most measures, even those with continuous options, in practice are used to generate binary yes/no estimates of exposure to types of violence. Some measures used higher scores to indicate higher severity. The few measures which calculate cut-off scores for severity of abuse included the CMQ and the CTQ. The ICAST manual stipulates that whether an act is abusive is dependent on context and legislation in that particular country and therefore researchers are urged to use an expert panel to determine what would be considered as abuse for each context in which the instrument is used.

##### Readability and comprehension (Supplement 4)

Reading age was only reported by a few studies. Flesch reading scores were calculated for the English–language versions of the measures where available. A Flesch score above 80 means the language is easily understood by an average 12-year-old school child, a score above 60 signifies the language is easily understood by 13- to 15-year-old school children (Flesch, 1948). All measures in this review that were scorable were in the 60–100 range. The following instruments scored above 80: APQ, CASRS, CEVQ, CTS-PC Picture, ETI-SF, GPBS, ICAST-C, KID-SAVE, ICAST-Trial C, JVQ, MPQ, PPS, PPI2, CNQ, TEQ, TISH, and SPaD.

##### Availability in multiple languages (Supplement 4)

Multiple translations have been published for measures commonly used in cross-country research. These include the ICAST, CTQ, APQ, CTS-PC, LITE-S, pediMACE, MACE, MICS, JVQ, MNBS, and ACE. The ICAST, APQ, MICS, ACE, and JVQ have been frequently used across low- and middle-income contexts and are available for researchers in many different languages.

## Discussion

This review systematically identified studies that utilized child self-report instruments for measuring violence exposure where some evaluation of psychometric properties was included. Thirty-nine original measures and 13 modified versions were described in 124 articles from across the world. Of these, most instruments were suitable for self-administration, with eight exclusively interviewer/clinician administered, and some interviewer administered for younger age groups. The measures’ methodological and psychometric quality as well as their administrative properties were carefully assessed. The aims of this review were to (1) identify standardized child self-report questionnaires on VAC; (2) describe their functionality, content, and structure; (3) evaluate their psychometric properties; and (4) provide evidence-based guidance in selecting an appropriate instrument for those wishing to conduct research on VAC.

The findings show that there is a large number of instruments to assess VAC, but that relatively few have been used multiple times and across different contexts. The most commonly used measures globally are the ACE, ICAST, JVQ, CTQ, MICS, and CTS-PC, or their modified versions.

The evidence base for the robustness of self-report questionnaires measuring VAC is rather limited, and further development and evaluation is urgently needed. Arguably, the lack of published research on the psychometric properties of these instruments is a historic and discipline-specific issue, with VAC only recently having been made a priority on the global public health agenda with the ratification of the Sustainable Development Goals. Prior to this, VAC research was limited to the fields of social work, pediatrics and psychology, predominantly with a focus on mental health outcomes.

### Considerations Relating to Content Validity

Content validity, arguably the most important measurement property, could only be assessed for nine measures. This is because many studies did not report on any content validity testing, and while it is assumed that many authors will do some pilot testing of instruments before deploying them in research, the methods and results of the pilot testing are rarely published. In order to not skew the overall content validity rating, we decided to only apply reviewer rating on content validity for the measures where some content validity data was available.

COSMIN criteria require assessment of relevance, comprehensiveness, and comprehensibility for content validity appraisal. Many of the studies did not assess all three at the same time or assessed these but did not provide in-depth description of the methodology used. Few studies on content validity were rated as high quality (*n* = 2). Small numbers of studies were rated sufficient in relation to relevance (*n* = 5), comprehensiveness (*n* = 4), and comprehensibility (*n* = 5). It should be noted that none of the studies in this review adhered to the rigorous guidance on content validity assessment developed by COSMIN. One of the COSMIN requirements for a sufficient rating in content validity mandates that studies make use of the target population, in this case children, to assess relevance, comprehensiveness and comprehensibility and not just professionals, but few studies do this. This limitation seems to be common in the development and adaptation of instruments that measure VAC.

### Considerations Relating to Structural Validity

Only 10 measures received an assessment of “sufficient” for structural validity. The fact that many widely used measures had “insufficient” structural validity or no structural validity testing at all is problematic. For most instruments in this systematic review, structural validity was not investigated, or was investigated through statistical techniques considered inadequate by COSMIN such as principal component analysis which automatically results in an insufficient rating for structural validity.

### Considerations Relating to Reliability

Test–retest reliability could only be assessed for 15 measures. Further, this was often conducted using correlations rather than Cohen’s kappa or ICCs which are required by COSMIN to achieve an “adequate” rating for study quality. It is also surprising that test–retest reliability was only assessed for 15 measures when there is a lively debate about the accuracy of recall in self-report measurement of VAC (Reuben et al., 2016), and test–retest reliability seems an easy way to assess stability of reporting.

Internal consistency could be assessed for 35 measures. Most researchers reported Cronbach’s alpha as a means to assess internal consistency, which is supported by COSMIN criteria despite the known difficulties and underlying assumptions when using alpha in assessing internal consistency (Sijtsma, 2009). Very few studies used more robust assessment methods such as McDonald’s Omega or structural equation modeling based methods. It is noted that few of the instruments were rated as having “sufficient” internal consistency and this is likely due to the nature and design of these measures as explained below. Further, instruments must have been judged to minimally have evidence of structural validity to receive a “sufficient” rating for internal consistency, excluding all studies from a high rating which did not test for structural validity.

Instruments that functioned as scales, such as the CTQ, have “sufficient” internal consistency because they were designed as a scale. Instruments that were designed as screening tools, for example, ICAST, JVQ, and others that cover multiple, different types of violence exposures received “insufficient” ratings. The COSMIN criterion for internal consistency should not be assessed for screening tools. This is because the items on screening tools for a single construct (e.g., physical violence) can be so inherently different that children who experience one or two items (e.g., smacking and hitting with a hard object) should not be assumed to also experience physical violence at the upper end of the spectrum (e.g., burning, choking, tying up, and assaulting with weapons) and therefore the measure is not assessing an underlying construct, but separate behaviors, which all fall within the definition of physical violence but may not occur in the same child throughout their lifespan. This highlights the need for subject specialists to be part of the review team when applying COSMIN criteria, as the criteria should be evaluated and applied to the specific characteristics of the phenomena assessed by each measure.

Notably, there were no studies that reported measurement error and only six studies investigated responsiveness/ability to detect change with very mixed results. More research is needed to establish the reliability of VAC measures.

### Considerations Relating to Construct Validity

Construct validity includes hypothesis testing, measurement invariance/cross-cultural validity, and criterion validity testing.

Hypothesis testing for construct validity assessed correlations or associations between the violence measure, mostly in dichotomized form and pre-specified health and behavioral outcomes. Study quality ratings for studies conducting hypothesis testing were generally high and the evidence for hypothesis testing “sufficient,” meaning that in general high correlations with the pre-specified outcomes across most measures were found.

Criterion validity was only assessed by one study, which compared a short version of the ICAST to the original ICAST. The body of evidence for this specific measure was rated as high and the criterion validity was assessed as “sufficient.” Considering that there were several modified and short versions of original measures included in this review, it is surprising that criterion validity was only assessed in one study.

As there is no gold-standard self-report VAC measure, criterion validity could not be assessed against a “gold standard” and while some studies assessed concurrence between different respondents, for example, parents and child or administrative records and child self-reports, these were considered too disparate in what they were assessing to meaningfully indicate criterion validity.

Measurement invariance across cultures/countries was assessed by only one study despite all of the more commonly used measures being widely deployed across low- and middle-income countries. There is an urgent need for research to address this because many of the prevalence estimates and reported risk factors and health outcomes are compared across countries without much evidence that the violence and risk factor/outcome measurements actually are invariant across settings.

There is little evidence of construct validity except for the least rigorous category of hypothesis testing and more research is urgently needed.

### Considerations Relating to Additional Items

Additional items measuring frequency, onset, duration, and severity of exposure as well as perpetrators, locations, disclosure, access to services, and participant burden may be important for individual researchers and are dependent on the aims and objectives of each study. Thus, some measures may be more suitable than others and some may require adaptation. It is important to note that the psychometric assessment conducted in this review focused predominantly on the screener questions, and not on the additional follow-up items related, for example, to perpetrators, settings, or disclosure factors, as none of the studies had conducted psychometric evaluations of contextual information.

### Considerations Relating to Practical Administration

Length, literacy requirements, accessibility, and copyright of measures should play an important role when selecting instruments. Many of the commonly deployed measures were suitable for reading age 12 + and easy to understand. For age groups younger than 10 years, developers recommend the use of interviewers in the administration of measures.

Most instruments had an average administration time of less than 30 minutes. Researchers must keep in mind that children and adolescents with violence exposure will require more time, and this must be taken into account in relation to participant burden.

Copyright and accessibility are a concern with one of the most used measures incurring relatively high costs for each application. For the CTQ and CTS-PC, access requires specific professional qualifications. However, the CTQ is one of the most rigorous instruments in terms of psychometric properties. Where possible, to promote accessibility and comparisons across contexts, we suggest trying to use open-access measures.

### Considerations Relating to Diversity

All except three measures included in this review were developed in HIC. While some measures, for example, the ICAST or MICS were specifically designed for use across contexts and settings and involved experts from across the globe in the development, little is known about the suitability of the measures for diverse populations, for example, ethnicity, language, sexual orientation, and whether there are types of VAC in specific settings of particular population groups which are overlooked by the developers of these measures. This is to an extent mitigated by the large number of validation studies in this review which used a measure developed in a HIC and translated and adapted it to a LMIC context; however, research is urgently needed to develop a conceptual framework of violence that is locally and internationally relevant and design an instrument that reliably and validly measures this concept of violence in children and adolescents.

### Strengths and Limitations of This Study

This review has several notable strengths and limitations. First, the comprehensiveness of the review is evidenced in the fact that an exhaustive range of databases was searched, and 20,429 abstracts screened. Second, the protocol for the review was pre-registered and inclusion and exclusion criteria were determined in advance. Third, 10% of all studies were double screened and extracted and all of the quality assessments were conducted by two reviewers. Fourth, the reporting on additional items and administration practicalities provides researchers with the necessary information to identify the most appropriate measure for use. Finally, the review included comprehensive and rigorous assessment of the quality of included studies and of the psychometric properties of included instruments following COSMIN 2018 criteria, which adds substantial credibility to the detailed assessment of measures expanding previous work (Saini et al., 2019).

There are several notable limitations of this review. First, the restriction to studies published in the English language precluded capture of studies published in other languages, and particularly those specifically developed for non-English speaking contexts. Second, there was a slight deviation from the COSMIN criteria in that we did not carry out rater content validity assessment for measures which did not at least have one study on content validity. We also introduced an additional assessment criterion on concurrence. Third, we included instruments that did not focus exclusively on VAC if they had a sub-scale that assessed some form of VAC. In these cases, we only assessed the psychometric properties of that sub-scale and not the properties of the whole measure, such as the APQ parental discipline sub-scale. Finally, we excluded any studies (n approx. 1900) that did not report on the psychometric properties of the measures they used. This resulted in the exclusion of some widely used measures, for example, those used in the VAC Surveys (Centers for Disease Control and Prevention, 2015), which are carried out across low- and middle-income countries and provide very valuable evidence on the prevalence and risk factors of children’s violence exposure. These should be assessed psychometrically in the future.

### Strengths and Limitations of the COSMIN Approach

This review used the 2018 COSMIN criteria which are stringent and complex to apply because of the multiple steps involved in the rating as described in the methods section. We found that some of the measures that received an inadequate score for their psychometric properties were quite close to the COSMIN established cut-off for an “sufficient” rating (e.g., coefficient alpha was .69 rather than 70 or above) and as such, we think the current COSMIN criteria provide an underestimation of the quality of the psychometric properties of an instrument. The same is true for the assessment of the methodological quality of studies which uses the “worst case counts” rule, and thus, down-rates the quality of a study for which there may be only a single concern with regards to study quality. This has also been found by Wittkowski et al. (2020) in their assessment of parent–infant attachment measures.

Further, as Wittkowski et al. (2020) and colleagues have previously reported, the assessment of measurement properties has rapidly developed over the past 10 years and some of the 2018 COSMIN criteria were not applicable to older studies. This included the use of confirmatory factor analysis, which was not common in articles published before 2010 using instead principal component analysis, which is automatically rated as inadequate for study quality and insufficient for psychometric properties. In this review, 47 included studies were published prior to 2010. Further, many older studies use correlational analysis to assess test–retest analysis instead of the COSMIN-required intra-class coefficients or Cohen’s kappa scores. These studies would have received an inadequate rating for study quality because of the nature of the statistical test applied, and an insufficient or indeterminate rating because the right test-statistics would not have been reported. Inclusion of older studies could have led to underestimation of the robustness of the psychometric properties, as older studies did less extensive psychometric testing than recent papers and would have been scored less highly as a result. Therefore, including newer studies might have indicated that the psychometric properties are more robust, than we have concluded from this review. It was, however, necessary to include older studies to give a broad picture of available VAC measures, many of which were developed prior to 2010 and are widely used in the field.

### Implications for Practice and Research

This review identified a lack of evidence for robust psychometric properties across a wide range of instruments to assess VAC. This is problematic because conclusions based on any of the measures will have inherent limitations. However, this does not mean that all these measures are flawed, rather that for many measures, there is no published evidence currently available or that the evidence which has been published has not been properly reported to meet the high standards of COSMIN criteria.

Some of these measures, in particular the ACE, ICAST, CTQ, CTS-PC, JVQ, and CECA have been used extensively and many have been recommended by, for example, the World Health Organization (Meinck et al., 2016). As with all measures deployed in research settings, researchers are advised to scrutinize each measure extensively to ensure it meets the aims and objectives of their research project and fits their purpose. Further research is needed on the less well evaluated psychometric properties of VAC measures such test–retest reliability, measurement error, responsiveness, structural validity, and measurement invariance to establish the psychometric properties and their robustness of the most commonly used measures. In particular, measures used across countries, cultures, languages, and population groups within a country should be assessed for cross-cultural validity to ensure that prevalence estimates and strength of correlations across population groups can be validly compared. This should be an important priority for future research on VAC measurement.

In addition, extensive qualitative work including cognitive interviewing and focus group discussions should be carried out with target populations to ensure the measure’s content validity for the target populations in relation to relevance, comprehensibility, and comprehensiveness. Researchers are encouraged to provide detailed information on the methodologies used and questions asked to help readers and reviewers to assess a study’s content validity. The results of any content validity testing should be published alongside other quantitative psychometrics gleaned from the main study as both will help build the body of research evidence on the robustness of measures.

As with all research on VAC, ethical considerations around child participation in measurement development, study planning, and interpretation should be incorporated as part of the research design, and strong protocols should be in place to deal with participant distress and reporting requirements.

## Conclusion

This is the first systematic review to employ the 2018 COSMIN criteria to assess the psychometric properties of child self-report VAC measures. A total of 39 measures and 13 modified versions were identified and evaluated. The methodological quality of the evidence for most measures was poor, and the psychometric properties were lacking in robustness. There is an urgent need for further research in this field. The review also provides a comprehensive assessment of a range of characteristics of each measure that researchers can weigh in deciding which instruments are most appropriate for their research questions and the children and caregivers with whom they conduct the studies.

## Critical Findings

• Most VAC child/adolescent self-report measures are not widely used and have little evidence for their psychometric rigor.
• A small number of instruments are widely used on diverse populations across many contexts using different translations.
• Little published evidence could be found for content validity, cross-cultural validity, structural validity, measurement error, and test–retest reliability in the widely used measures.
• The ICAST, ACE, CTQ, CTS-PC, CECA, and JVQ have sufficient psychometric properties to be used in research.

## Practice, Policy, and Research implications

• Research on structural validity, test–retest reliability, and cross-cultural validity of VAC child self-report measures is urgently needed.
• Extensive in-depth qualitative research involving cognitive interviews is necessary to determine content validity and cultural sensitivity of measures.
• Researchers must ensure measures are appropriate and relevant for their local context through extensive piloting and adaptation with the target population.

## Supplemental Material

sj-pdf-1-tva-10.1177_15248380221082152 – Supplemental Material for Measuring Violence Against Children: A COSMIN Systematic Review of the Psychometric Properties of Child and Adolescent Self-Report Measures Click here for additional data file. Supplemental Material, sj-pdf-1-tva-10.1177_15248380221082152 for Measuring Violence Against Children: A COSMIN Systematic Review of the Psychometric Properties of Child and Adolescent Self-Report Measures by Lakshmi Neelakantan, Bridget Steele, Janina Jochim, Lynn M. Davies, Mark Boyes, Jane Barlow and Michael Dunne in Trauma, Violence, & Abuse.

## References

[bibr1-15248380221082152] Bhat D. P. Singh M. Meena G. S. (2012). Screening for abuse and mental health problems among illiterate runaway adolescents in an Indian metropolis. Archives of Disease in Childhood, 97(11), 947–951. 10.1136/archdischild-2011-301603 22904267

[bibr4-15248380221082152] Centers for Disease Control and Prevention . (2015). Violence against children surveys (VACS). http://www.cdc.gov/violenceprevention/vacs/index.html

[bibr5-15248380221082152] Essau C. A. Sasagawa S. Frick P. J. (2006). Psychometric properties of the alabama parenting questionnaire. Journal of Child and Family Studies, 15(5), 595–614. 10.1007/s10826-006-9036-y

[bibr6-15248380221082152] Fang X. Fry D. A. Brown D. S. Mercy J. A. Dunne M. P. Butchart A. R. Corso P. S. Maynzyuk K. Dzhygyr Y. Chen Y. McCoy A. Swales D. M. (2015). The burden of child maltreatment in the East Asia and Pacific region. Child Abuse & Neglect, 42(3), 146–162. 10.1016/j.chiabu.2015.02.012. 25757367 PMC4682665

[bibr7-15248380221082152] Finkelhor D. Ji K. Mikton C. Dunne M. (2013). Explaining lower rates of sexual abuse in China. Child Abuse & Neglect, 37(10), 852–860. 10.1016/j.chiabu.2013.07.006. 23958110

[bibr8-15248380221082152] Flesch R. (1948). A new readability yardstick. Journal of Applied Psychology, 32(3), 221–233. 10.1037/h0057532. 18867058

[bibr10-15248380221082152] Hamby S. Finkelhor D. Kopiec K. (2000). Asking children about victimization: A qualitative study of the language of victimization surveys. In Victimization of Children and Youth: An International Research Conference. Crimes Against Children Research Centre University of New Hampshire.

[bibr11-15248380221082152] Hecker T. Radtke K. M. Hermenau K. Papassotiropoulos A. Elbert T. (2016). Associations among child abuse, mental health, and epigenetic modifications in the proopiomelanocortin gene (POMC): A study with children in Tanzania. Development and Psychopathology, 28(4pt2), 1401–1412. 10.1017/S0954579415001248. 26753719

[bibr13-15248380221082152] Hughes K. Bellis M. A. Hardcastle K. A. Sethi D. Butchart A. Mikton C. Jones L. Dunne M. P. (2017). The effect of multiple adverse childhood experiences on health: A systematic review and meta-analysis. The Lancet. Public Health, 2(8), Article e356–e366. 10.1016/S2468-2667(17)30118-4. 29253477

[bibr41-15248380221082152] Laurin J. Wallace C. Draka J. Aterman S. J. Tonmyr L. (2018). Youth self-report of child maltreatment in representative surveys: a systematic review. Health Promot Chronic Dis Prev Can, 38(2), 37–54. 10.24095/hpcdp.38.2.01. 29443484 PMC5833635

[bibr14-15248380221082152] Malik F. D. Shah A. A. (2007). Development of child abuse scale: Reliability and validity analyses. Psychology and Developing Societies, 19(2), 161–178. 10.1177/097133360701900202.

[bibr15-15248380221082152] Mathews B. Pacella R. Dunne M. P. Simunovic M. Marston C. (2020). Improving measurement of child abuse and neglect: A systematic review and analysis of national prevalence studies. Plos One, 15(1), Article e0227884. 10.1371/journal.pone.0227884. 31990913 PMC6986759

[bibr16-15248380221082152] Meinck F. Boyes M. E. Cluver L. Ward C. L. Schmidt P. DeStone S. Dunne M. P. (2018). Adaptation and psychometric properties of the ISPCAN Child Abuse Screening Tool for use in trials (ICAST-Trial) among South African adolescents and their primary caregivers. Child Abuse & Neglect, 82, 45–58. 10.1016/j.chiabu.2018.05.022. 29860107

[bibr17-15248380221082152] Meinck F. Steinert J. I. Sethi D. Gilbert R. Bellis M. A. Mikton C. Alink L. Baban A. (2016). Measuring and monitoring national prevalence of child maltreatment: A practical handbook. World Health Organization Regional Office for Europe.

[bibr18-15248380221082152] Mohammadkhani P. Mohammadi M. R. Nazari M. A. Salavati M. Razzaghi O. M. (2003). Development, validation and reliability of child abuse self-report scale (CASRS) in Iranian students. Medical Journal of the Islamic Republic of Iran, 17(1), 51–58.

[bibr20-15248380221082152] Mokkink L. B. de Vet H. Prinsen C. Patrick D. L. Alonso J. Bouter L. M. Terwee C. B. (2018). COSMIN risk of bias checklist for systematic reviews of patient-reported outcome measures. Quality of Life Research: An International Journal of Quality of Life Aspects of Treatment, Care and Rehabilitation, 27(5), 1171–1179. 10.1007/s11136-017-1765-4. 29260445 PMC5891552

[bibr21-15248380221082152] Mokkink L. B. Prinsen C. A. C. Patrick D. L. Alonso J. Bouter L. M. deVet H. C. W. Terwee C. B. (2018). COSMIN methodology for systematic reviews of patient-reported outcome measures (PROMS): User manual. VU University Medical Center.

[bibr22-15248380221082152] Moore S. E. Scott J. G. Ferrari A. J. Mills R. Dunne M. P. Erskine H. E. Devries K. M. Degenhardt L. Vos T. Whiteford H. A. McCarthy M. Norman R. E. (2015). Burden attributable to child maltreatment in Australia. Child Abuse & Neglect, 48, 208–220. 10.1016/j.chiabu.2015.05.006. 26056058

[bibr23-15248380221082152] Nordstrom-Klee B. A. (2001). Violence exposure and child behavioral and emotional problems: The moderating role of maternal acceptance. Thesis. Wayne State University.

[bibr24-15248380221082152] O’Boyle J. V. (2002). The role of abuse-related and disclosure events in moderating the impact of child sexual abuse. Thesis. University of Missouri.

[bibr25-15248380221082152] Page M. J. McKenzie J. E. Bossuyt P. M. Boutron I. Hoffmann T. C. Mulrow C. D. Shamseer L. Tetzlaff J. M. Akl E. A. Brennan S. E. Chou R. Glanville J. Grimshaw J. M. Hróbjartsson A. Lalu M. M. Li T. Loder E. W. Mayo-Wilson E. McDonald S. McGuinness LA Moher D. (2021). The PRISMA 2020 statement: An updated guideline for reporting systematic reviews. BMJ, 372, n71. 10.1136/bmj.n71. 33782057 PMC8005924

[bibr26-15248380221082152] Prinsen C. Mokkink L. B. Bouter L. M. Alonso J. Patrick D. L. de Vet H. Terwee C. B. (2018). COSMIN guideline for systematic reviews of patient-reported outcome measures. Quality of Life Research: An International Journal of Quality of Life Aspects of Treatment, Care and Rehabilitation, 27(5), 1147–1157. 10.1007/s11136-018-1798-3. 29435801 PMC5891568

[bibr42-15248380221082152] Reuben A. Moffitt T. E. Caspi A. Belsky D. W. Harrington H. Schroeder F. Hogan S. Ramrakha S. Poulton R. Danese A. (2016). Lest we forget: comparing retrospective and prospective assessments of adverse childhood experiences in the prediction of adult health. J Child Psychol Psychiatry, 57(10), 1103–1112. 10.1111/jcpp.12621. 27647050 PMC5234278

[bibr27-15248380221082152] Ritacco G. Suffla S. (2012). A critical review of child maltreatment indices: Psychometric properties and application in the South African context. African Safety Promotion Journal, 10(2), 3–17. http://hdl.handle.net/10500/8913.

[bibr28-15248380221082152] Saini S. M. Hoffmann C. R. Pantelis C. Everall I. P. Bousman C. A. (2019). Systematic review and critical appraisal of child abuse measurement instruments. Psychiatry Research, 272, 106–113. 10.1016/j.psychres.2018.12.068. 30580133

[bibr29-15248380221082152] Shirinbayan P. Salavati M. Soleimani F. Saeedi A. Asghari-Jafarabadi M. Hemmati S., and Vameghi R. (2020). The psychometric properties of the Persian version of childhood experience of care and abuse questionnaire (CECA). Galen Medical Journal, 9, Article e1663. 10.31661/gmj.v9i0.1663. PMC8344028 34466564

[bibr30-15248380221082152] Sierau S. White L. O. Klein A. M. Manly J. T. von Klitzing K. Herzberg P. Y. (2018). Assessing psychological and physical abuse from children’s perspective: Factor structure and psychometric properties of the picture-based, modularized child-report version of the parent-child conflict tactics scale - revised (CTSPC-R). Plos One, 13(10), Article e0205401. 10.1371/journal.pone.0205401. 30296298 PMC6175525

[bibr31-15248380221082152] Sijtsma K. (2009). On the use, the misuse, and the very limited usefulness of Cronbach's alpha. Psychometrika, 74(1), 107–120. 10.1007/s11336-008-9101-0. 20037639 PMC2792363

[bibr32-15248380221082152] Silveira A. L. d. S. Grassi-Oliveira R. (2016). Semantic validation of the ISPCAN child abuse screening tools (ICAST) in Brazilian Portuguese. Trends in Psychiatry and Psychotherapy, 38(2), 105–110. 10.1590/2237-6089-2016-0012. 27409137

[bibr33-15248380221082152] Terwee C. B. Prinsen C. Chiarotto A. Westerman M. J. Patrick D. L. Alonso J. Bouter L. M. de Vet H. Mokkink L. B. (2018). COSMIN methodology for evaluating the content validity of patient-reported outcome measures: A Delphi study. Quality of Life Research: An International Journal of Quality of Life Aspects of Treatment, Care and Rehabilitation, 27(5), 1159–1170. 10.1007/s11136-018-1829-0. 29550964 PMC5891557

[bibr34-15248380221082152] Tonmyr L. Draca J. Crain J. Macmillan H. L. (2011). Measurement of emotional/psychological child maltreatment: A review. Child Abuse & Neglect, 35(10), 767–782. 10.1016/j.chiabu.2011.04.011. 22018520

[bibr35-15248380221082152] Walsh C. Jamieson E. MacMillan H. Trocmé N. (2004). Measuring child sexual abuse in children and youth. Journal of Child Sexual Abuse, 13(1), 39–68. 10.1300/J070v13n01_03. 15353376

[bibr36-15248380221082152] Walsh C. A. MacMillan H. L. Trocmé N. Jamieson E. Boyle M. H. (2008). Measurement of victimization in adolescence: Development and validation of the childhood experiences of violence questionnaire. Child Abuse & Neglect, 32(11), 1037–1057. 10.1016/j.chiabu.2008.05.003. 18992940

[bibr39-15248380221082152] Wittkowski A. Vatter S. Muhinyi A. Garrett C. Henderson M. (2020). Measuring bonding or attachment in the parent-infant-relationship: A systematic review of parent-report assessment measures, their psychometric properties and clinical utility. Clinical Psychology Review, 82, 101906. 10.1016/j.cpr.2020.101906. 32977111 PMC7695805

[bibr40-15248380221082152] Yoon S. Speyer R. Cordier R. Aunio P. Hakkarainen A. (2020). A systematic review evaluating psychometric properties of parent or caregiver report instruments on child maltreatment: Part 2: Internal consistency, reliability, measurement error, structural validity, hypothesis testing, cross-cultural validity, and criterion validity. Trauma, Violence & Abuse, 22(5), 1296–1315. 10.1177/1524838020915591. PMC8739544 32270753

