# Supplementary material for: Measuring Violence Against Children: A COSMIN Systematic Review of the Psychometric Properties of Child and Adolescent Self-Report Measures
Source: Trauma Violence Abuse. 2022 Apr 21;24(3):1832–47. doi: 10.1177/15248380221082152 (PMC10240621; doi:10.1177/15248380221082152)
Supplement: sj-pdf-1-tva-10.1177_15248380221082152 – Supplemental Material for Measuring Violence Against Children: A COSMIN Systematic Review of the Psychometric Properties of Child and Adolescent Self-Report Measures [file sj-pdf-1-tva-10.1177_15248380221082152.pdf]

Contents

Supplement 1: Search terms 2
Supplement 2: List of included studies 4
Supplement 3: Overview of included measures 12
Supplement 4: Overview of measures and access requirements 19
Supplement 5: Methodological quality content validity studies 25
Supplement 6: Quality of content validity studies 26
Supplement 7: Overall content validity quality rating 27
Supplement 8: Methodological quality of psychometric studies 28
Supplement 9: Quality of psychometric properties 35
Supplement 10: Overall quality rating of psychometric properties 41

Supplement 1: Search terms

Embase (1974 – present)
Global Health (1973 – present)
OVID MEDLINE (R) – all
PsychInfo (1806 – present)

Search Number	Search
#1	(child adj3 abuse).ab,kw,ti
#2	(child adj3 maltreatment).ab,kw,ti
#3	(adolescen* adj3 abuse).ab,kw,ti.
#4	(teen* adj3 abuse).ab,kw,ti.[3]
#5	(youth adj3 abuse).ab,kw,ti.[3]
#6	(young adj adult* adj3 abuse).ab,kw,ti.[3]
#7	(child* adj3 sexual adj abuse).ab,kw,ti.
#8	(child* adj3 sexual adj harassment).ab,kw,ti. [3,8]
#9	(child* adj3 sexual adj molest*).ab,kw,ti. [3,8]
#10	(child* adj3 rape).ab,kw,ti.
#11	(violen* adj3 against adj3 children).ab,kw,ti.
#12	(child* adj3 victim*).ab,kw,ti.
#13	(corporal adj punishment).ab,kw,ti.
#14	(harsh adj punishment).ab,kw,ti [3]
#15	(physical adj punishment).ab,kw,ti. [3]
#16	(adverse adj childhood adj experiences).ab,kw,ti.
#17	(harsh adj discipline). ab,kw,ti. [3]
#18	(physical adj discipline).ab,kw,ti. [3]
#19	(harsh adj parenting).ab,kw,ti
**#20 [Forms of abuse]**	**#1 OR #2 OR #3 OR #4 OR #5 OR #6 OR #7 OR #8 OR #9 OR #10 OR #11 OR #12 OR #13 OR #14 OR #15 OR #16 OR #17 OR #18#19**
**#21 [4] [Type of measure]**	**(measur* OR scale* OR instrument* OR questionnaire* OR survey* OR inventor* OR tool* or interview*).af**
**#22 [Psychometric Properties]**	**(psychometric* OR factor* OR reliabilit* OR consistenc* OR validit* OR sensitivity OR specificity OR predict*).af**
**#23 [Existing Scales]**	((Adverse adj Childhood adj Experiences) OR (ACE) OR (Abusive adj Sexual adj Exposure adj Scale) OR (ASES) OR (Child adj Abuse adj2 Neglect adj Inventory adj Schedule adj1 revised) OR (CANIS-R) OR (Cuestinaro adj2 Evaluacion adj2 Maltrato adj2 Ninos adj2 Discapacidad) OR (CEMND) OR (Child adj Maltreatment adj History adj1 Self adj Report) OR (CMHSR) OR (Child adj Maltreatment adj Interview adj Schedule adj1 Short adj Form) OR (CMIS-SF) OR (Childhood adj experiences adj2 violence adj questionnaire) OR (CEVQ) OR (Childhood adj experiences adj2 care adj2 abuse) OR (CECA) OR (Dimensions adj2 Discipline) OR (DDI) OR (International adj Association adj3 Prevention adj2 Child adj Abuse adj2 Neglect adj Child adj Abuse adj Screening adj Tool) OR (ICAST) OR (Child adj Abuse adj Screening adj Tool) OR (ICAST-P) OR (ICAST-R) OR (ICAST-C) OR (ICAST-CI) OR (ICAST-CH) OR (Juvenile adj Victimization adj Questionnaire)

	OR (JVQ) OR (NorVold adj Abuse adj Questionnaire) OR (NorAQ) OR (Alabama OR Parenting OR Questionnaire) OR (APQ) OR (Adverse adj Childhood adj Experiences adj1 International adj Questionnaire) OR (ACE adj IQ) OR (Child adj Physical adj Maltreatment) OR (CPM) OR (Childhood adj Trauma adj Questionnaire) OR (CTQ) OR (Multidimensional adj Neglectful adj Behaviour adj Scale) OR (MNBS) OR (Parent adj Child adj Conflict adj Tactics adj Scale) OR (PC adj CTS) OR (Sexual adj Abuse adj Interview adj Schedule) OR (Sexual adj Experiences adj Survey) OR (SES) OR (Adult adj Adolescent adj Parenting adj Inventory adj 2) OR (AAPI adj 2) OR (Child adj abuse adj potential adj inventory) OR (CAPI) OR (Brief adj Child adj Abuse adj Potential adj Inventory) OR (BCAPI) OR (Parenting adj2 Family adj adjustment adj scale) OR (PAFAS) OR (Child adj Abuse adj Inventory) OR (CAI) OR (Abuse adj Dimensions adj Inventory) OR (ADI) OR (Child adj Abuse adj2 Trauma adj Scale) OR (CAT) OR (Child adj Exposure adj2 Violence adj Form) OR (CEVF) OR (Sexual adj Abuse adj Severity adj Score) OR (SASS)).af.[5]
#24	#21 OR # 22
#25	24 AND 23
**RESULT #26**	**#20 AND #25**[6]

PROQUEST

Search Number	Search
#1	(child NEAR abuse) OR (child NEAR maltreatment) OR (corporal NEAR punishment) OR (harsh NEAR discipline) (OR violence NEAR against NEAR children). Ti,ab
#2	(psychometric* OR validity OR reliability), af
#3	#1 AND #2

Social Sciences Citation Index

Search Number	Search
#1	TS=(child near abuse)
#2	TS=(child near maltreatment)
#3	TS=(violence NEAR against NEAR children)
#4	TS=(corporal NEAR punishment)
#5	TS=(harsh NEAR discipline)
#6	#1 OR #2 OR #3 OR #4 OR #5
#7	TI=(psychometric* OR validity OR reliability OR sensitivity OR specificity)
#8	TI=(measure* OR scale* OR Instrument* OR questionnaire OR survey OR inventor* OR interview)
#9	#6 AND #7 AND #8

Supplement 2: List of included studies

Ajdukovic, M., Susac, N., & Rajter, M. (2013). Gender and age differences in prevalence and incidence of child sexual abuse in Croatia. *Croatian Medical Journal*, *54*(5), 469–479.

Baniasad, M., Noghani, F., & Gerami, M. (2016). Comparison of mental health in students with and without experience of child abuse. *Acta Medica Mediterranea*, Vol. 32, pp. 2115–2120.

Barnes, J. E., Noll, J. G., Putnam, F. W., & Trickett, P. K. (2009). Sexual and physical revictimization among victims of severe childhood sexual abuse. *Child Abuse & Neglect*, Vol. 33, pp. 412–420.

Bernstein, D. P., Ahluvalia, T., Pogge, D., & Handelsman, L. (1997). Validity of the childhood trauma questionnaire in an adolescent psychiatric population. *Journal of the American Academy of Child and Adolescent Psychiatry*, *36*(3), 340–348.

Betancourt, T. S., Ng, L. C., Kirk, C. M., Munyanah, M., Mushashi, C., Ingabire, C., … Sezibera, V. (2014). Family-based prevention of mental health problems in children affected by HIV and AIDS: an open trial. *Special Issue: Children Born into Families Affected by HIV.*, Vol. 28, pp. S359–S368. R

Beyazit, U., & Ayhan, A. B. (2019). A Study on the Mother Education Program for the Prevention of Child Neglect: *122*(6), 2178–2200.

Bhat, D. P., Singh, M., & Meena, G. S. (2012). Screening for abuse and mental health problems among illiterate runaway adolescents in an Indian metropolis. *Archives of Disease in Childhood*, *97*(11), 947–951.

Boonmann, C., Grisso, T., Guy, L., Colins, O., Mulder, E., Vahl, P., … Doreleijers, T. (2016). Childhood traumatic experiences and mental health problems in sexually offending and non-sexually offending juveniles. *Child and Adolescent Psychiatry and Mental Health*, Vol. 10, p. 45. R

Brockie, T. N., Dana-Sacco, G., Wallen, G. R., Wilcox, H. C., & Campbell, J. C. (2015). The Relationship of Adverse Childhood Experiences to PTSD, Depression, Poly-Drug Use and Suicide Attempt in Reservation-Based Native American Adolescents and Young Adults. *American Journal of Community Psychology*, *55*(3–4), 411–421.

Burton, D. L. (2008). An exploratory evaluation of the contribution of personality and childhood sexual victimization to the development of sexually abusive behavior. *Sexual Abuse: Journal of Research and Treatment*, pp. 102–115.

Cecil, C. A. M., McCrory, E. J., Viding, E., Holden, G. W., M.;, B. C. A., & Barker, E. D. (2016). Initial Validation of a Brief Pictorial Measure of Caregiver Aggression: The Family Aggression Screening Tool. *Assessment*, *23*(3), 307–320.

Chang, H.-Y., Lin, C.-L., Chang, Y.-T., Tsai, M.-C., & Feng, J.-Y. (2013). Psychometric testing of the Chinese version of ISPCAN Child Abuse Screening Tools Children's Home Version (ICAST-CH-C). *Children and Youth Services Review*, *35*(12), 2135–2139.

Charak, R., Byllesby, B. M., Roley, M. E., Claycomb, M. A., Durham, T. A., Ross, J., … Jana, E. (2016). Latent classes of childhood poly-victimization and associations with suicidal behavior among adult trauma victims: Moderating role of anger. *Child Abuse & Neglect*, Vol. 62, pp. 19–28.

Chung, M. C., & Chen, Z. S. (2017). Child Abuse and Psychiatric Co-morbidity Among Chinese Adolescents: Emotional Processing as Mediator and PTSD from Past Trauma as Moderator. *Child Psychiatry and Human Development*, *48*(4), 610–618.

Cole, D. A., Martin, N. C., Sterba, S. K., Sinclair-McBride, K., Roeder, K. M., Zelkowitz, R., … R., Z. (2014). Peer victimization (and harsh parenting) as developmental correlates of cognitive reactivity, a diathesis for depression. *Journal of Abnormal Psychology*, pp. 336–349.

Crooks, C. V, Scott, K., Ellis, W., Wolfe, D. A., & A., W. D. (2011). Impact of a universal school-based violence prevention program on violent delinquency: Distinctive benefits for youth with maltreatment histories. *Child Abuse & Neglect*, *35*(6), 393–400.

Crowley, T. J., Mikulich, S. K., Ehlers, K. M., Hall, S. K., & Whitmore, E. A. (2003). Discriminative Validity and Clinical Utility of an Abuse-Neglect Interview for Adolescents With Conduct and Substance Use Problems, *160*(8), 1461–1469.

Cuevas, C. A., Finkelhor, D., Ormrod, R., & Turner, H. (2009). Psychiatric diagnosis as a risk marker for victimization in a national sample of children. *Journal of Interpersonal Violence*, *24*(4), 636–652.

de la Vega, A., de la Osa, N., Ezpeleta, L., Granero, R., & Domenech, J. M. (2011). Differential effects of psychological maltreatment on children of mothers exposed to intimate partner violence. *Child Abuse & Neglect*, Vol. 35, pp. 524–531.

de Zoysa, P., Newcombe, P. A., & Rajapakse, L. (2006). Corporal punishment in the Sri Lankan context: Psychological outcomes for our children. *New Developments in Parent-Child Relations.*, pp. 1–40.

Devries, K. M., Knight, L., Allen, E., Parkes, J., Kyegombe, N., & Naker, D. (2017). Does the Good Schools Toolkit Reduce Physical, Sexual and Emotional Violence, and Injuries, in Girls and Boys equally? A Cluster-Randomised Controlled Trial. *Prevention Science*, 1–15.

Dias, A., Sales, L., Hessen, D. J., & Kleber, R. J. (2015). Child maltreatment and psychological symptoms in a Portuguese adult community sample: the harmful effects of emotional abuse. *European Child & Adolescent Psychiatry*, *24*(7), 767–778.

Dingwall, J. A. (1997). *Perception of facial expressions of emotion by children with severe, moderate and low levels of physical discipline.* (ProQuest Information & Learning).

Donovan, K. L. (2009). Trajectories of maternal verbal aggression across the early adolescent years: What patterns are most harmful to low -income community adolescents? (Columbia University).

dos Santos, D. J. F., & Alberto, I. M. M. (2016). Developing a family risk assessment tool in adolescent offenders: An exploratory study. *Child & Adolescent Social Work Journal*, Vol. 33, pp. 103–113.

Dubowitz, H., Villodas, M. T., Litrownik, A. J., Pitts, S. C., Hussey, J. M., Thompson, R., … Runyan, D. (2011). Psychometric properties of a youth self-report measure of neglectful behavior by parents. *Child Abuse & Neglect*, *35*(6), 414–424.

Edwards, A. A. R. (2014). Youth Reports of Psychological Maltreatment, Social Anxiety, and Aggression: Evaluating Rejection Sensitivity as a Mediator (Columbia University).

Esposito, C. L., & Clum, G. A. (2002). Social support and problem-solving as moderators of the relationship between childhood abuse and suicidality: Applications to a delinquent population. *Journal of Traumatic Stress*, Vol. 15, pp. 137–146.

Essau, C., Satoko, S., & Frick, P. (2006). Psychometric Properties of the Alabama Parenting Questionnaire. *Journal of Child and Family Studies*, *15*(5), 597–616.

Everson, M. D., Smith, J. B., Hussey, J. M., English, D., Litrownik, A. J., Dubowitz, H., … E.D., K. (2008). Concordance between adolescent reports of childhood abuse and child protective service determinations in an at-risk sample of young adolescents. *Child Maltreatment*, *13*(1), 14–26.

Feng, J. Y., Chen, C. C., Chang, Y. T., Chang, H. Y., & Shih, C. L. (2020). A psychometric analysis of a short form of the Chinese version of the ISPCAN child abuse screening tools – Children's home version (SC-ICAST-CH) using multidimensional item response theory. *Child Abuse & Neglect*, *109*, 104693.

Finkelhor, D, Omrod, R., & Turner, H. (2005). Measuring poly-victimization using the Juvenile Victimization Questionnaire. *Child Abuse and Neglect*, Vol. 29, pp. 1297–1312.

Finkelhor, David, Hamby, S. L., Ormrod, R., & Turner, H. (2005). The Juvenile Victimization Questionnaire: Reliability, validity, and national norms. *Child Abuse and Neglect*, *29*(4), 383–412.

Finkelhor, David, Shattuck, A., Turner, H., & Hamby, S. (2013). Improving the Adverse Childhood Experiences Study Scale. *JAMA Pediatrics*, *167*(1), 70.

Flowers, A. L., Hastings, T. L., & Kelley, M. L. (2000). Development of a Screening Instrument for Exposure to Violence in Children: The KID-SAVE. *Journal of Psychopathology and Behavioral Assessment 2000 22:1*, *22*(1), 91–104.

Fuller-Thomson, E., Baird, S. L., Dhrodia, R., & Brennenstuhl, S. (2016). The association between adverse childhood experiences (ACEs) and suicide attempts in a population-based study. *Child: Care, Health and Development*, *42*(5), 725–734.

Gidycz, C., & Koss, M. (1989). The impact of adolescent sexual victimization: standardized measures of anxiety, despression and behavioral deviancy. *Violence and Victims*, *4*(2), 139–149.

Gillick, T. A. (1997). Depression in adolescent female victims of intrafamilial child sexual abuse.

Greenwald, R., & Rubin, A. (1999). Assessment of Posttraumatic Symptoms in Children: Development and Preliminary Validation of Parent and Child Scales: *Research on Social Work Practice*, *9*(1), 61–75.

Halgunseth, L. C., Espinosa-Hernandez, G., Joeng, H.-K., Chang, Y., Card, N., & Reid, A. (2017). Parenting and adolescent adjustment in Mexico: Initial evidence of validity for the Mexican Parenting Questionnaire for Adolescents (MPQ-A). *Journal of Child and Family Studies*, Vol. 26, pp. 471–481.

Hamby, S., Finkelhor, D., & Kopiec, K. (2000). Asking children about victimization: a qualitative study of hte language of victimization surveys. *Victimization of Children and Youth: An International Research Conference*.

Hernandez-Guzman, L., Montesinos, M. G., Bermudez-Ornelas, G., Freyre, M.-A., & Alcazar-Olan, R. J. (2013). Parental Practices Scale for Children. *Revista Colombiana de Psicologia*, Vol. 22, pp. 151–161.

Hilker, K. A. (2002). Somatic complaints and health care utilization in children exposed to violence (Louisiana State University and Agricultural & Mechanical College).

Horesh, N., Nachshoni, T., Wolmer, L., & Toren, P. (2009). A comparison of life events in suicidal and nonsuicidal adolescents and young adults with major depression and borderline personality disorder. *Comprehensive Psychiatry*, *50*(6), 496–502.

Horesh, N., Ratner, S., Laor, N., & Toren, P. (2008). A comparison of life events in adolescents with major depression, borderline personality disorder and matched controls: a pilot study. *Psychopathology*, *41*(5), 300–306.

Hosseinkhani, Z., Nedjat, S., Aflatouni, A., Mahram, M., & Majdzadeh, R. (2015). Socioeconomic inequality and child maltreatment in Iranian schoolchildren. *Eastern Mediterranean Health Journal*, *21*(11), 819–827.

Isele, D. (2016). *The role of Adverse Childhood Experiences (ACEs in clinical disorders: A new assessment tool and evaluation of links with borderline personalitz symptoms* (Universität Konstanz).

Kaufman Kantor, G., Holt, M. M. K., Mebert, C. C. J., Straus, M. A. M., Drach, K. K. M., Ricci, L. L. R., … Brown, W. (2004). Development and Preliminary Psychometric Properties of the Multidimensional Neglectful Behavior Scale-Child Report. *Child Maltreatment*, *9*(5), 409–428.

Kidman, R., Smith, D., Piccolo, L. R., & Kohler, H. P. (2019). Psychometric evaluation of the Adverse Childhood Experience International Questionnaire (ACE-IQ) in Malawian adolescents. *Child Abuse and Neglect*, *92*, 139–145.

Kjellgren, C., Svedin, C. G., & Nilsson, D. (2013). Child Physical Abuse-Experiences of combined

treatment for children and their parents: A pilot study. *Special Issue: Physical Abuse in High Risk Families*, Vol. 19, pp. 275–290.

Kobulsky, J. M., Holmes, M. R., Yoon, S., & Perzynski, J. M. (2016). Physical abuse after child protective services investigation and adolescent substance use. *Children and Youth Services Review*, Vol. 71, pp. 36–44.

Kobulsky, J. M., Kepple, N. J., Holmes, M. R., & Hussey, N. J. (2017). Concordance of parent- and child-reported physical abuse following child protective services investigation. *Child Maltreatment*, Vol. 22, pp. 24–33.

Kolko, D., Kazdin, A., & Day, B. (1996). Children's Perspectives in the Assessment of Family Violence: Psychometric Characteristics and Comparison to Parent Reports. *Child Maltreatment*, *1*(2), 156–167.

Krischer, M. K., & Sevecke, K. (2008). Early traumatization and psychopathy in female and male juvenile offenders. *Special Issue: Psychopathic Traits and Risk Assessment in Children and Adolescents*, *31*(3), 253–262.

Lang, J., & Connell, C. (2017). Development and validation of a brief trauma screening measure for children: The Child Trauma Screen. *Psychological Trauma : Theory, Research, Practice and Policy*, *9*(3), 390–398.

Lang, J., & Connell, C. (2018). The Child Trauma Screen: A Follow-Up Validation. *Journal of Traumatic Stress*, *31*(4), 540–548. h

Leguizamo, A. (2000). Juvenile sex offenders: An object relations approach. *Dissertation Abstracts International: Section B: The Sciences and Engineering*, p. 1641.

Lev-Wiesel, R., & Zohar, G. (2014). The role of dissociation in self-injurious behavior among female adolescents who were sexually abused. *Journal of Child Sexual Abuse*, *23*(7), 824–839.

Levendosky, A., & Huth-Bocks, A. (2002). Adolescent peer relationships and mental health functioning in families with domestic violence. *Journal of Clinical Child and Adolescent Psychology*, Vol. 31, pp. 206–218.

Lindhiem, O., Shaffer, A., & Kolko, D. J. (2014). Quantifying discipline practices using absolute versus relative frequencies: clinical and research implications for child welfare. *Journal of Interpersonal Violence*, *29*(1), 66–81.

Lipschitz, D. S., Bernstein, D. P., Winegar, R. K., & Southwick, S. M. (1999). Hospitalized adolescents' reports of sexual and physical abuse: A comparison of two self-report measures. *Journal of Traumatic Stress*, *12*(4), 641–654.

Lupis, A. A. (2015). Inter-generational transmission of trauma in Croatia: Relational anger and guilt in children of war veterans (Long Island University, The Brooklyn Center).

Maciel, M. R., Mello, A. F., Fossaluza, V., Nobrega, L. P., Cividanes, G. C., Mari, J. J., & Mello, M. F. (2013). Children working on the streets in Brazil: predictors of mental health problems. *European Child & Adolescent Psychiatry*, *22*(3), 165–175.

Malik, F. D., & Shah, A. A. (2007). Development of child abuse scale: Reliability and validity analyses. *Psychology and Developing Societies*, *19*(2), 161–178.

Matsuura, N., Hashimoto, T., & Toichi, M. (2009). Correlations among self-esteem, aggression, adverse childhood experiences and depression in inmates of a female juvenile correctional facility in Japan: Regular article. *Psychiatry and Clinical Neurosciences*, *63*(4), 478–485.

Meinck, F., Cosma, A. P., Mikton, C., & Baban, A. (2017). Psychometric properties of the Adverse Childhood Experiences Abuse Short Form (ACE-ASF) among Romanian high school students. *Child Abuse and Neglect*, *72*.

Meinck, F, Boyes, M., Cluver, L., Ward, C., Schmidt, P., DeStone, S., & Dunne, M. (2018). Adaptation and Psychometric Properties of the ISPCAN Child Abuse Screening Tool for use in Trials (ICAST-TRIAL) Among South African Adolescents and their Primary Caregivers. *Child Abuse & Neglect*.

Meinck, Franziska, Murray, A. L., Dunne, M. P., Schmidt, P., Nikolaidis, G., Petroulaki, K., … Browne, K. (2020). Measuring violence against children: The adequacy of the International Society for the Prevention of Child Abuse and Neglect (ISPCAN) child abuse screening tool - Child version in 9 Balkan countries. *Child Abuse & Neglect*, *108*, 104636.

Melmed, L. R. (2012). Cognitive Style as a Mediator between Parental Psychological Maltreatment and Depression in Adolescent Boys (Columbia University).

Miller, E. A., Green, A. E., Fettes, D. L., & A, A. E. (2011). Prevalence of maltreatment among youths in public sectors of care. *Child Maltreatment*, Vol. 16, pp. 196–204.

Mizuki, R., & Fujiwara, T. (2020). Validation of the Japanese version of the Childhood Trauma Questionnaire-Short Form (CTQ-J). *Psychological Trauma : Theory, Research, Practice and Policy*.

Mohammadkhani, P., Mohammadi, M., Nazari, M., Slvati, M., & Razzaghi, O. (2003). Development, validation and reliability of child abus self report scale (CASRS) in Iranian students. *Medical Journal of the Islamic Republic of Iran*, *17*(1), 51–58.

Mohler-Kuo, M., Landolt, M. A., Maier, T., Meidert, U., Schönbucher, V., & Schnyder, U. (2014). Child sexual abuse revisited: A population-based cross-sectional study among swiss adolescents. *Journal of Adolescent Health*, *54*(3), 304–311.

Munoz, D. T. (1996). Child sexual abuse in a sample of male and female Hispanic and White nonclinical adolescents: Extending the reliability and validity of the Trauma Symptom Inventory (TSI) (University of Southern California).

Mikaeili, N., Barahmand, U. & Abdi, R. (2013). The prevalence of different kinds of child abuse and the characteristics that differentiate abused from nonabused male adolescents. *Journal of Interpersonal Violence*, *28*(5), 975–996.

Negriff, S., Schneiderman, J. U., & Trickett, P. K. (2017). Concordance between self-reported childhood maltreatment versus case record reviews for child welfare-affiliated adolescents: Prevalence rates and associations with outcomes. *Child Maltreatment*, Vol. 22, pp. 34–44.

Nilsson, D., Gustafsson, P., & Svedin, C. (2010). Self-reported potentially traumatic life events and symptoms of post-traumatic stress and dissociation. *Nordic Journal of Psychiatry*, *64*(1), 19–26.

Nooner, K. B. (2007). *Latent Class Analysis of New Self-Report Measures of Physical and Sexual Abuse*.

Nordstrom-Klee, B. A. (2001). Violence exposure and child behavioral and emotional problems: The moderating role of maternal acceptance. Wayne State University, Detroit, MI, US.

O’Boyle, J. V. (2002). The role of abuse-related and disclosure events in moderating the impact of child sexual abuse. University of Missouri, St Louis, MO, US.

Ometto, M., de Oliveira, P. A., Milioni, A. L., Dos Santos, B., Scivoletto, S., Busatto, G. F., … Cunha, P. J. (2016). Social skills and psychopathic traits in maltreated adolescents. *European Child & Adolescent Psychiatry*, *25*(4), 397–405.

Park, S. (2018). Reliability and Validity of the Early Trauma Inventory Self Report-Short Form among Korean Adolescents. *Journal of the Korean Academy of Child and Adolescent Psychiatry*, *29*(1), 2.

Peh, C. X., Shahwan, S., Fauziana, R., Mahesh, M. V, Sambasivam, R., Zhang, Y., … Subramaniam, M. (2017). Emotion dysregulation as a mechanism linking child maltreatment exposure and self-harm behaviors in adolescents. *Child Abuse and Neglect*, *67*, 383–390.

Penelo, E., Viladrich, C., & Domenech, J. M. (2012). Adolescents' perceptions of parental behavior: psychometric properties of the short Egna Minnen Betraffande Uppfostran-Adolescent version (S-EMBU-A) in a clinical sample. *Comprehensive Psychiatry, 53*(1), 87–94.

Puffer, E. S., Annan, J., Sim, A. L., Salhi, C., & Betancourt, T. S. (2017). The impact of a family skills training intervention among Burmese migrant families in Thailand: A randomized controlled trial. *PloS One, 12*(3), e0172611.

Richters, J., & Martinez, P. (1993). The NIMH community violence project: II. Children's distress symptoms associated with violence exposure. *Psychiatry, 56*, 7–21.

Sahaimi, M. F., Pa, M. N. M., & Taib, F. (2020). A Malay Version of the International Society for the Prevention of Child Abuse and Neglect Screening Tool for Children: A Study of Its Validity and Reliability. *The Malaysian Journal of Medical Sciences : MJMS, 27*(4), 97.

Salamone, C. (2006). *Examining the parent perception inventory within the context of behaviour parent training*. Auburn University.

Schaeffer, C. M., Swenson, C. C., Tuerk, E. H., & Henggeler, S. W. (2013). Comprehensive treatment for co-occurring child maltreatment and parental substance abuse: Outcomes from a 24-month pilot study of the MST-Building Stronger Families program. *Child Abuse & Neglect*, Vol. 37, pp. 596–607.

Schneider, W., MacKenzie, M., Waldfogel, J., & Brooks-Gunn, J. (2015). Parent and Child Reporting of Corporal Punishment: New Evidence from the Fragile Families and Child Wellbeing Study. *Child Indicators Research, 8*(2), 347–358.

Seiler, A., Kohler, S., Ruf-Leuschner, M., & Landolt, M. A. (2016). Adverse childhood experiences, mental health, and quality of life of Chilean girls placed in foster care: An exploratory study. *Psychological Trauma: Theory, Research, Practice, and Policy*, Vol. 8, pp. 180–187.

Sesar, K., & Zivcic-Becirevic, I. (2008). Multi-type maltreatment in childhood and psychological adjustment in adolescence: Questionnaire study among adolescents in Western Herzegovina Canton. *Croatian Medical Journal*, Vol. 49, pp. 243–256.

Shaffer, A., Lindhiem, O., & Kolko, D. J. (2012). Treatment effects of a modular intervention for early-onset child behavior problems on family contextual outcomes. *Journal of Emotional and Behavioral Disorders*, Vol. 21, pp. 277–288.

Shamu, S., Gevers, A., Mahlangu, B., Shai, P., & Chirwa, E. (2016). Prevalence and risk factors for intimate partner violence among grade 8 learners in urban South Africa: Baseline analysis from the Skhokho supporting success cluster randomised controlled trial. *International Health*, Vol. 8, pp. 18–26.

Shirinbayan P, Salvati, M., Soleimani, F., Saeedi, A., Asghari-Jafarabadi, M., Hemmati, S., & Vameghi, R. (2020). The Psychometric Properties of the Persian Version of Childhood Experience of Care and Abuse Questionnaire (CECA). *Galen Medical Journal, 9*, e1663.

Sierau, S., White, L. O., Klein, A. M., Manly, J. T., von Klitzing, K., & Herzberg, P. Y. (2018). Assessing psychological and physical abuse from children's perspective: Factor structure and psychometric properties of the picture-based, modularized child-report version of the Parent-Child Conflict Tactics Scale – Revised (CTSPC-R). *PLOS ONE, 13*(10), e0205401.

Silveira, A. L. da S., & Grassi-Oliveira, R. (2016). Semantic validation of the ISPCAN child abuse screening tools (ICAST) in Brazilian Portuguese. *Trends in Psychiatry and Psychotherapy, 38*(2), 105–110.

Skillings, J. L. (2006). The Scale of Parental Discipline: Assessing the reliability and validity of a new measurement scale (The University of Toledo).

Smith, M. S., Lindsey, C. R., & Hansen, C. E. (2006). Corporal Punishment and the Mediating Effects of Parental Acceptance-Rejection and Gender On Empathy in a Southern Rural Population. *Cross-*

Cultural Research: The Journal of Comparative Social Science, pp. 287–305.

Sofuoglu, Z., Saryer, G., & Ataman, M. G. (2016). Child Maltreatment in Turkey: Comparison of Parent and Child Reports. *Central European Journal of Public Health*, Vol. 24, pp. 217–222.

Soler, L., Kirchner, T., Paretilla, C., & Forns, M. (2013). Impact of Poly-Victimization on Mental Health: The Mediator and/or Moderator Role of Self-Esteem. *Journal of Interpersonal Violence*, *28*(13), 2695–2712.

Stewart, C., Kirisci, L., Long, A. L., & Giancola, P. R. (2015). Development and Psychometric Evaluation of the Child Neglect Questionnaire. *Journal of Interpersonal Violence*, *30*(19), 3343–3366.

Stoltz, J.-A. M., Shannon, K., Kerr, T., Zhang, R., Montaner, J. S., & Wood, E. (2007). Associations between childhood maltreatment and sex work in a cohort of drug-using youth. *Social Science and Medicine*, Vol. 65, pp. 1214–1221.

Hecker, T., Hermenau, K., Salmen, C., Teicher M. & Elbert, T. (2016). Harsh discipline relates to internalizing problems and cognitive functioning: Findings from a cross-sectional study with school children in Tanzania. *BMC Psychiatry*, Vol. 16, p. 2.

Tanaka, M., Wekerle, C., Leung, E., Waechter, R., Gonzalez, A., Jamieson, E., & MacMillan, H. L. (2012). Preliminary evaluation of the childhood experiences of violence questionnaire short form. *Journal of Interpersonal Violence*, *27*(2), 396–407. https://doi.org/10.1177/0886260511416462

Tanaka, M., Wekerle, C., Schmuck, M. Lou, & Paglia-Boak, A. (2011). The linkages among childhood maltreatment, adolescent mental health, and self-compassion in child welfare adolescents. *Child Abuse and Neglect*, *35*(10), 887–898.

Tangeman, K. (2004). Trauma and aggression in male juvenile delinquents (California State University, Long Beach).

Tardif-Williams, C. Y., Tanaka, M., Boyle, M. H., & MacMillan, H. L. (2015). The Impact of Childhood Abuse and Current Mental Health on Young Adult Intimate Relationship Functioning:

Tingskull, S., Svedin, C. G., Agnafors, S., Sydsjö, G., deKeyser, L., & Nilsson, D. (2015). Parent and Child Agreement on Experience of Potential Traumatic Events. *Child Abuse Review*, *24*(3), 170–181.

Tucker, M. C., & Rodriguez, C. M. (2014). Utility of Child Report: Correspondence with Parent-Reported Child Abuse Risk. *Child Abuse Review*, *23*(5), 334–341.

Turner, H. A., Finkelhor, D., & Ormrod, R. (2010). Poly-victimization in a national sample of children and youth.

Turner, H. A., Vanderminden, J., Finkelhor, D., Hamby, S., & Shattuck, A. (2011). Disability and victimization in a national sample of children and youth. *Child Maltreatment*, Vol. 16, pp. 275–286.

Ungar, J. (2004). Coping styles and early experiences of victimization in sexually abusive and delinquent youth (University of Windsor (Canada)).

Usta, J., Farver, J. M., & Danachi, D. (2013). Child maltreatment: The Lebanese children's experiences. *Child: Care, Health and Development*, *39*(2), 228–236.

Van Leeuwen, K. G., & Vermulst, A. (2004). Some Psychometric Properties of the Ghent Parental Behavior Scale. *European Journal of Psychological Assessment*, pp. 283–298.

van Vugt, E., Lanctot, N., Paquette, G., Collin-Vezina, D., & Lemieux, A. (2014). Girls in residential care: From child maltreatment to trauma-related symptoms in emerging adulthood. *Child Abuse & Neglect*, Vol. 38, pp. 114–122.

Walsh, C. A., MacMillan, H. L., Trocmé, N., Jamieson, E., & Boyle, M. H. (2008). Measurement of victimization in adolescence: development and validation of the Childhood Experiences of Violence

Questionnaire. *Child Abuse & Neglect*, *32*(11), 1037–1057.

Wekerle, C., Wolfe, D., Lynn Hawkins, D., Pittman, A., Glickman, A., & Lovald, B. (2001). Childhood maltreatment, posttraumatic stress symptomology, and adolescent dating violence: Considering the value of adolescent perceptions of abuse and a trauma mediational model. *Development and Psychopathology*, *13*, 847–871.

Wolfe, D. A., Scott, K., Wekerle, C., & Pittman, A. L. (2001). Child maltreatment: risk of adjustment problems and dating violence in adolescence. *Journal of the American Academy of Child and Adolescent Psychiatry*, *40*(3), 282–289.

Xing, X., Zhang, H., Shao, S., & Wang, M. (2017). Child negative emotionality and parental harsh discipline in Chinese preschoolers: The different mediating roles of maternal and paternal anxiety. *Frontiers in Psychology*, Vol. 8.

Zimmerman, P. A. (2006). Adolescent mothers: Youth in need of developmentally appropriate services (Portland State University).

Zlomke, K., Bauman, S., & Lamport, D. (2015). Adolescents' perceptions of parenting behavior: Validation of the Alabama Parenting Questionnaire adolescent self report. *Journal of Child and Family Studies*, Vol. 24, pp. 3159–3169.

Zolotor, A. J., Runyan, D. K., Dunne, M. P., Jain, D., Péturs, H. R., Ramirez, C., … Isaeva, O. (2009). ISPCAN Child Abuse Screening Tool Children's Version (ICAST-C): Instrument development and multi-national pilot testing. *Child Abuse & Neglect*, *33*, 833–841.

Supplement 3: Overview of included measures

Instrument (Study authors)	Measure retrievable	Constructs	Subscales	Recall period	Target population	Number of items	Range of score	Response options	Locations	Perpetrators	Disclosure	Frequency	Severity	Trivialisation questions
**Multiple forms of violence**														
Adverse Childhood Experiences Adolescent Short Form (ACE-ASF)	Yes	Physical Abuse; Emotional Abuse; Sexual Abuse	Physical abuse, emotional abuse, sexual abuse	Lifetime	18+ (sometimes used for adolescents)	8	*	5 point Likert scale	No	No	No	Yes	No	No
Adverse Childhood Experiences International Questionnaire (ACE-IQ)	Yes	Child exposure to trauma	Emotional; physical; sexual abuse and emotional and physical neglect	Lifetime	18+ (sometimes used for adolescents)	14	*	5 point Likert scale	No	Yes	No	Yes	No	No
Adverse Childhood Experiences (ACE)	Yes	Child abuse and household dysfunction	Emotional; physical; sexual abuse and emotional and physical neglect	Lifetime	18+ (sometimes used for adolescents)	14	*	5 point Likert scale	No	Yes	No	Yes	No	No
Modified Adverse Childhood Experiences (M-ACE)	Yes	Child abuse and household dysfunction	Emotional; physical; sexual abuse and emotional and physical neglect	Lifetime	18+ (sometimes used for adolescents)	14	0-14	Yes or no	No	No	No	*	No	No
Child Abuse Scale (CAS1)	Yes	Child abuse	Psychological abuse, physical abuse, emotional neglect, physical neglect	*	Children aged 8-12	34	0-3	Never to always	No	No	No	Yes	No	*
Child Abuse Self-report Scale (CASRS)	Yes	Child abuse	Emotional Abuse, physical abuse, sexual abuse, physical neglect	*	*	38	0-2	Never to always	No	No	No	Yes	Yes	*
Child Abuse Survey (CAS2)	No	Child abuse	Physical and sexual abuse	Lifetime	Adolescents	*	0-6	Never-more than 20 times	No	Yes	No	Yes	Yes	*
Child Maltreatment Interview Schedule Short Form (CMIS-SF)	Yes	Child maltreatment and household dysfunction	Witnessing IPV, psychological unavailability of parents, psychological abuse, physical abuse, sexual abuse	Lifetime	Children	10	0-1 sexual abuse, 0-6 other abuse	Yes/no for sexual abuse, never – over 20 times for other types of abuse	No	Yes	No	Yes	Yes	*
Child Maltreatment Questionnaire (CMQ)	No	Child maltreatment history	Physical abuse, emotional abuse, sexual abuse,	Lifetime	Adolescents	*	*	Measures frequency but not reported how	No	Yes	No	Yes	Yes	*

			witnessing DV, physical neglect											
Child Trauma Screen (CTS)	Yes	Childhood trauma and symptoms	Trauma, symptoms	Lifetime	Children aged 7-17	10 (4 exposure, 6 reaction)	0-1 trauma, 4-point Likert scale reaction	Yes or no (trauma), never - 3+ times/week	No	No	No	No	Yes	*
Childhood Experiences of Violence Questionnaire (CEVQ)	Yes	Childhood violence exposure	Items on bullying, witnessing domestic violence, emotional abuse, corporal punishment, physical abuse, sexual abuse	Lifetime	Adolescents	16 screeners	0-5 for all abuse, 0-1 sexual	Never-more than 10 times all abuse, sexual abuse yes/no and then 4 items on frequency	No	Yes	Yes	Yes	Yes	No
Childhood Experiences of Care and Abuse (CECA)	Yes	Childhood maltreatment, neglect, antipathy, physical abuse, sexual abuse	Parental care (neglect and antipathy), physical abuse, sexual abuse	Lifetime	11-14	20	*	5-point Likert scale	No	Yes	No	Yes	Yes	No
Childhood Trauma Questionnaire (CTQ)	Yes	Childhood trauma	Sexual, physical, emotional abuse and neglect	Lifetime	12+	70	1-5 per item	Never true, rarely true, sometimes true, often true, very often true	*	*	*	Yes	Yes	Yes
Childhood Trauma Questionnaire Short Form (28 item) (CTQ-SF)	Yes	Childhood trauma exposure	Physical abuse, physical neglect, emotional abuse, emotional neglect, sexual abuse	Lifetime	adults and adolescents	28	5-point Likert scale	Never true - very often true	No	No	No	Yes	Yes	Yes
Child Maltreatment History Self-Report (CMH-SR)	Yes	Physical abuse, sexual abuse	Physical abuse, sexual abuse	Lifetime	Adolescents – used in children aged 6-18	7	0-3	Never-often	No	No	No	Yes	*	*
Colorado Adolescent Rearing Inventory (CARI)	Yes	Child abuse history	Physical, psychological and sexual abuse and neglect	Lifetime	13-19	50	0-1	Yes/no, onset, duration, frequency	No	Yes	No	Yes	Yes	No
Comprehensive Trauma Interview (CTI2)	Yes	Maltreatment experiences and other childhood trauma	Sexual abuse, physical abuse, emotional abuse and neglect, witnessing DV	Lifetime	Adolescents	22 screeners plus follow-ups	0-1	Yes or no, onset, frequency, duration	No	Yes	Yes	Yes	Yes	No
Early Trauma Inventory	Yes	Childhood	Physical abuse,	Lifetime	Adolescents	27	0-1	Yes or no	No	No	No	No	No	No

Short Form (ETISR-SF)		trauma	emotional abuse, sexual abuse, general traumatic experiences											
Family Aggression Screening Tool (FAST)	Yes	Caregiver aggression	Emotional, verbal and physical abuse and exposure to emotional, verbal and physical IPV	Lifetime	Children	12 pictorial representations	0-1, 0-10	Yes or no, ongoing, frequency	No	Yes	No	Yes	No	No
International Society for the Prevention of Child Abuse and Neglect Child Abuse Screening Tool (ICAST-CH)	Yes	Child abuse in home. Has been revised in V3 to also include institution and 35 items	Physical violence, psychological violence, Sexual violence, Exposure to violence (DV, community violence).	Past year and lifetime	Children aged 11-17	38	*	Never, has happened but not in the past year, once or twice this year, several times this year, about once a month, more than once a month, every week	No	Yes	No	Yes	*	*
International Society for the prevention of Child Abuse and Neglect Child Abuse Screening Tool (ICAST-CI)	Yes	Child abuse in institutions (note this instrument no longer exists, the ICAST C was modified in V3 and now contains institutional abuse as an answer option)	Physical, emotional, sexual abuse	Past year and lifetime	Children aged 11-17	44	*	Never, has happened but not in the past year, once or twice this year, several times this year, about once a month, more than once a month, every week	Yes	Yes	No	Yes	No	No
International Society for the Prevention of Child Abuse and Neglect Child Abuse Screening Tool (ICAST-Trial C)	Yes	Child abuse	Physical, emotional and sexual abuse and neglect	Past month	Children aged 10-18	27	0-8 per item (0-243)	0-8+ times	No	No	No	Yes	No	No
International Society for the Prevention of Child Abuse and Neglect Child Abuse Screening Tool Chinese Short Form (SC-ICAST-CH)	Yes	Child abuse	Physical, emotional and sexual abuse and neglect	Past year and lifetime	Children aged 12-18	22	0-7 per item	Never, has happened but not in the past year, once or twice this year, several times this year, about once a month, more than once a month, every week	No	No	No	Yes	No	No
Juvenile Victimization Questionnaire (JVQ)	Yes	Exposure to violence and	Conventional crime; child maltreatment	Past year and	Youth (ages 8-17)	34	0-34	Yes, no, number of times it happened	Yes	Yes	Yes	Yes	Yes	No

		violence related themes	(sexual, physical, and emotional; peer and sibling victimisation; sexual victimisation; witnessing and indirect victimisation	lifetime										
Children Screen for Adolescent Violence Exposure (KID-SAVE)	Yes	Violence exposure	Traumatic violence, indirect violence and physical/verbal abuse	Lifetime	children grades 3-7	34	0-2	never, sometimes, a lot	No	no	no	Yes	Yes	No
Life Incidence of Traumatic Events self-report (LITE)	Yes	Traumatic events	Non-interpersonal events and interpersonal events including abuse	Lifetime	Adolescents	16	0-1	Yes or no, frequency, onset, distress, distress at present	No	No	No	Yes	Yes	*
Longitudinal Studies on Child Abuse and Neglect (LONGSCAN)	Yes	Child abuse	Physical abuse; sexual abuse; emotional abuse	Lifetime	Age 12	30	*	Yes or no	No	Yes	Yes	No	*	No
Maltreatment and Abuse Chronology of Exposure - Pediatric Version (PediMACE)	Yes	Child maltreatment	Emotional abuse, physical abuse, sibling violence, neglect, witnessing violence, peer violence, sexual violence and parental loss	Lifetime	Children	45	0-1	Yes or no, also measures onset, burden as feeling helpless or terrified	No	No	No	Yes	Yes	No
Maltreatment and Abuse Chronology of Exposure (MACE)	Yes	Childhood maltreatment	Verbal abuse, emotional abuse, physical maltreatment, sexual abuse, witnessing IPV, witnessing violence against siblings, peer verbal abuse, peer physical bullying, emotional neglect and physical abuse	Lifetime	Adults, but has been used with children aged 6-17	75	0-1	Yes or no, onset	No	Yes	No	Yes	Yes	No
Parent-Child Conflict Tactics Scale - Revised (Picture-based child-report) (CTS-PC-P)	Yes	Physical and emotional abuse by caregiver	NA	Past year	4-16 years (picture cards provided to children aged 4-8)	22	0-4	Did not occur-every time	No	Yes	No	Yes	No	No
Parent-Child Conflict Tactics Scales (Child	Yes	Physical and emotional abuse	Psychological aggression, Physical	Past year	Children	22	0-7	Never-more than 20 times and not in the	No	Yes	No	Yes	Yes	No

Version) (CTS-PC)		by caregiver	assault, severe assault, very severe assault, non-violent discipline					past year, but happened before						
Parental Practices Scale (PPS)	Yes	Parental practices	Rejection; punishment; responsiveness; warmth; support;	Lifetime	Children	27	*	5-point Likert scale	No	Yes	No	Yes	No	No
Parent Perception Inventory (PPI2)	Yes	Parenting practices	Harsh/critical (only subscale used in studies)	Lifetime	Children	36 (total) 18 (harsh/ critical parenti ng subscal e)	18- 90	4- or 5-point Likert scale	No	Yes	No	Yes	No	No
Structured Interview of Family Assessment Risk (SIFAR)	No	Tool for assessing adolescent offenders' family risk	13 areas of family life (physical health, mental health, substance abuse, education, employment, housing/transport, legal problems, violence, ethnic and social dissonance, poverty, social net, social security and parenting)	Lifetime	Adolescent offenders and their parents	*	*	Answers rated on a scale of increasing risk (0-5)	Yes	Yes	No	Yes	Yes	No
Traumatic Events Questionnaire (TEQ)	No	Child and sexual abuse and other types of trauma/violence	Child physical abuse and child sexual abuse	Lifetime	Adolescents	49	4-28	7-point Likert scale	No	No	No	Yes	yes	No
Traumatic Events Screening Inventory (TESI-C)	Yes	Trauma exposure	Child physical abuse; sexual abuse; emotional abuse	Lifetime	Adolescents (15+)	18	*	Yes/no/unsure/refus e	No	Yes	No	Yes	Yes	No
Things I have seen and heard (TISH)	No	Exposure to violence and violence related themes	sexual violence, community violence	Lifetime	Children aged 6-8	15	*	5-point Likert scale	No	No	No	Yes	*	No
							**Only sexual violence**							
Checklist of sexual abuse	Yes	Sexual abuse and	Sexual abuse events,	*	Age 5-12 –	63	0-1	Yes or no	No	Yes	Yes	No	No	No

and related events (young child) (C-SARS)		events related to the abuse	sexual abuse related events, disclosure related events		children who experienced sexual abuse									
Child Sexual Abuse Questionnaire (CSAQ1)	Yes	Child sexual abuse	Non-contact sexual abuse, contact sexual abuse	Lifetime	Adolescents 12+	15	0-1 non-contact, 0-2 contact	Non-contact: yes no, Contact: no, yes someone tried and didn't succeed, yes someone tried and succeeded	Yes	Yes	Yes	No	No	*
Childhood Sexual Abuse Questionnaire (CSAQ2)	Yes	Child sexual abuse	Contact and non-contact sexual abuse	*	Adolescents	17	*	*	*	*	Yes	No	*	*
Sexual Abuse Exposure Questionnaire (SAEQ)	Yes	Sexual acts before the age of 16	Onset of sexual abuse; duration; severity; perpetrator; trust for perpetrator	Lifetime	*	10	*	Yes or no	No	Yes	Yes	No	Yes	No
Sexual Experiences Survey (SES)	Yes	Sexual victimization	*	3 months-5 years	Adolescents and adults	5	*	Yes or no	*	*	*	Yes	No	No
**Only psychological violence**														
Comprehensive Assessment of Psychological Maltreatment - Child Version (CAPM)	No	Psychological maltreatment	Spurning, terrorizing, exploiting and corrupting, denying emotional responsiveness, isolating, mental health, medical and educational neglect	Past year	11-18	34	1-4	Never- almost always	No	Yes	No	Yes	*	*
**Only physical violence**														
Alabama Parenting Questionnaire (APQ)	Yes	5 parenting behaviours	Corporal punishment, involved parenting, positive parenting, inconsistent discipline, and poor monitoring	Current self-report	6-18	42 (3 items on corporal punishment)	0-4	Never to always	No	Yes	No	Yes	No	No
Ghent Parental Behaviour Scale (GPBS)	Yes	Parenting behaviour	Parental involvement, monitoring, discipline including harsh punishment, positive reinforcement, problem solving	*	Children	55	0-4	Never to always	No	Yes	No	Yes	No	*

Mexican Parenting Questionnaire: Adolescent Version (MPQ)	Yes	Parenting behaviour	Affection, verbal guidance, monitoring, communication, physical and verbal punishment	Current self-report	Adolescents	13	1-5	5 items: I strongly disagree-I strongly agree; 8 items: almost never - almost always	No	Yes	No	No	No	No
Multiple Indicator Cluster Survey (MICS)	Yes	Harsh discipline and punishment	Physical punishment	Lifetime	Children	*	*	*	No	Yes	No	Yes	No	No
Scale of Parental Discipline (SPaD)	Yes	Physical punishment and assessment of whether it was fair or not	Three primary scales (fairness, frequency, specific frequency) and ten subscales	Lifetime	Children	120	*	4- or 5-point Likert scale	No	Yes	No	Yes	No	No
Schedule for the Assessment of IPV Exposure in Children (SAIPVEC)	No	Physical maltreatment	NA	Lifetime	Children	8	*	*	No	Yes	No	*	*	No
Schedule for Risk Factors (SRF)	No	Harsh discipline	Discipline and adult supervision	Lifetime	Children aged 8-18	15	0-60	4-point Likert Scale	*	*	*	Yes	*	*
Violence History Questionnaire (VHQ)	No	History of violence	discipline, corporal punishment and physical abuse	*	Typically for adults but adjusted for children	12	0-84	7-point Likert scale (never to at least once a week)	No	Yes	No	Yes	*	*
					**Only Neglect**									
Child Neglect Questionnaire (CNQ)	Yes	Child neglect	Physical, emotional, education and supervision neglect	Past 6 months	Children aged 10-12	46	5-1	Never to always	No	No	No	Yes	No	No
Multidimensional neglectful behavior scale (MNBS-CR)	Yes	Child neglect	Physical, emotional, cognitive and supervisory neglect; depression, failure to protect the child, alcohol use, inclination for socially acceptable responses and general neglect	Lifetime	Children 10-15	2 sets of 66 picture cards	0-49	Choice between two picture cards	No	Yes	No	YEs	No	No

* no information available

Supplement 4: Overview of measures and access requirements

Instrument (Study authors)	Copyright status	Cost	Reading age	Flesch reading score	Mode of application	Time to complete	Languages	User handbook	Participant burden	Regulatory agency's requirement for use
**Multiple forms of violence**										
Adverse Childhood Experiences Adolescent Short Form (ACE-ASF)	Open access (citation required)	Free	11+	77.6	Pen and paper	~40 minutes	Romanian, English	No	No	None
Adverse Childhood Experiences International Questionnaire (ACE-IQ)	Open access (citation required)	Free	11+	77.6	Tablets with trained interviewers to read out loud in local language or self-completion	*	chiChichewa; English	Yes	No	None
Adverse Childhood Experiences (ACE)	Open access (citation required)	Free	18+	77.6	Tablets or pen and paper	*	English	Yes	No	None
Modified Adverse Childhood Experiences (M-ACE)	Open access (citation required)	Free	NA	77.6	Phone call- interview	*	English	Yes	No	None
Child Abuse Scale (CAS1)	Open access (citation required)	Free	8-12 years	60.7	*	*	Urdu, English	*	*	*
Child Abuse Self-report Scale (CASRS)	Open access (citation required)	Free	*	80.6	Paper-pencil	*	English, Farsi	*	*	*
Child Abuse Survey (CAS2)	*	*	12+	Not retrievable	Self-completion	*	English	*	*	*
Child Maltreatment Questionnaire (CMQ)	*	*	* used with adolescents 15+	65.7	Self-completion	*	Croatian	*	*	*
Child Maltreatment Interview Schedule Short Form (CMIS-SF)	Open access (citation required)	Free	11+	59.3	Self-completion	*	English	*	*	*
Child Trauma Screen (CTS)	online from https://www.chdi.org/	Free	7+	76.4	self-completion, telephone or in person interview	5-10 minutes	English	Yes	No	Professionals working with children
Childhood	Request to use	Free	12+	72.9	Self-completion paper-	40-120 minutes	Persian, English	Yes	No	None

Experiences of Care and Abuse (CECA)					pencil questionnaire sent to participants homes					
Childhood Experiences of Violence Questionnaire (CEVQ)	Part of paper	Free	*	89.1	Self-completion	*	English	*	Yes	*
Childhood Trauma Questionnaire Short Form (28 item) (CTQ-SF)	Copyrighted	£175.49 for manual and 25 score sheets, pack of 25 score sheets £64.49	14+	66.1	Self-completion	5 minutes	English	Yes	No	People certified by a professional organisation recognised by Pearson Assessment
Childhood Trauma Questionnaire (CTQ)	Copyrighted	Paid; available from Pearson Assessments	12+	66.1	Face to face or self-completion with paper and pencil	*	English	Yes	*	Qualified professional required for administration
Child Maltreatment History Self-Report (CMH-SR)	Open access (citation required)	Free	12+	78.5	Interviewer-guided	10-15 minutes	Hindi, English	*	*	*
Colorado Adolescent Rearing Interview (CARI)	Open access (citation required)	Free	12+	65.9	Interview	20-45 minutes	English	Yes	No	None
Comprehensive Trauma Interview (CTI2)	Open access (citation required)	Free	*	76.9	Interviewer led	*	English	Yes	No	None
Early Trauma Inventory Short Form (ETISR-SF)	Contact Douglas Bremner	$1,0000 for 1000 applications	12+	84.8	Self-completion	*	English	Yes	No	*
Family Aggression Screening Tool (FAST)	Request to use	Free	*- computer applied so reading not a requirement	(Mostly pictures) 78.4	Computer assessment with pictures	5 minutes	English	*	No	None
Children Screen for Adolescent Violence Exposure (KID-SAVE)	Part of paper	Free	Flesh Kincaid fourth grade	80.3 (for the ICAST-C)	Self-completion with interviewer instruction - read out loud for younger children	*	English	*	*	*
ICAST-CH	Request to use from ISPCAN	Free (ICAST CI and ICAST CH combined into ICAST-C	11+	80.3 (for the ICAST-C)	Self-completion paper-pencil questionnaire and or tablet, has also been used with	~30 minutes	English, Arabic, Turkish, Portuguese, Croatian, Chinese, Farsi, Malay	Yes	Yes	None

		V3)			interviewers					
ICAST-CI	Request to use from ISPCAN	Note ICAST-CI no longer exists	11+	80.3	Self-completion paper-pencil questionnaire and or tablet, has also been used with interviewers	~ 30 minutes	Luganda, English	Yes	Yes	None
ICAST-Trial C	Open access (citation required)	Free	10+	80.3	Tablets or paper, also used with ACASI and with interviewers	~ 30 minutes	Xhosa, English	No	No	None
SC-ICAST-CH	Open access (citation required)	Free	11+	Interview (no reading)	Self-completion with paper and pencil	~ 15 minutes	Mandarin	No	No	None
Juvenile Victimization Questionnaire (JVQ)	Open access (citation required)	Free	10+	85.1	Self-administered, interviewer-administered, computer-administered typically, but also over the telephone and paper versions. The choice of the version will also influence mode of administration i.e. challenging to administer the full version without a computer.	20-30 minutes (shorter versions available)	English, Spanish, Chinese, Urdu	Yes	Yes	Any experienced examiner can administer, administration for para-professionals under supervision
Life Incidence of Traumatic Events self-report (LITE)	Ricky Greenwald rg@childtrauma.com	Free	Grade 3 and higher	74.3	Self-completion or for younger children with interviewer guidance, face-to-face or telephone interview also possible	5 minutes	English, Swedish, Spanish, Persian, German	Yes	No	*
Longitudinal Studies on Child Abuse and Neglect (LONGSCAN)	Copyrighted	*	12	78.5	Self-completion paper-pencil questionnaire	*	English	Yes	No	None
Maltreatment and Abuse Chronology of Exposure - Pediatric Version (pediMACE)	Part of paper	*	7+	63.1	interview by mental health professionals	45 minutes	German, English, Swahili	Yes, as part of thesis	No	clinicians
Maltreatment and Abuse Chronology of Exposure (MACE)	Open access (citation required)	Free	*	63.1	Self-completion but with children interviewer-led	*	English, German, Spanish	Yes	No	*

Parent-Child Conflict Tactics Scale - Revised Picture-based child-report (CTS-PC Picture)	Copyrighted	CTSPC £122.40 for handbook and 10 autoscore forms	Interviewer applied, reading age probably 10+	100	Interviewer led	*	German, English	Yes	No	People certified by a professional organisation recognised by Pearson Assessment
Parent-Child Conflict Tactics Scales (CTS-PC Child Version)	Copyrighted	CTSPC £122.40 for handbook and 10 autoscore forms	11+	78.8	Self-completion or interviewer led	*	English	Yes	No	People certified by a professional organisation recognised by Pearson Assessment
Parental Practices Scale (PPS)	Open access (citation required)	Free	01-Jun	100	Self-completion	*	Spanish	*	No	*
Parent Perception Inventory (PPI2)	Open access (citation required)	Free	6+	98.3	Self-completion	*	English	*	No	*
Structured Interview of Family Assessment Risk (SIFAR)	available from developers	free	NA	Not retrievable	Interview (must be conducted by experienced psychologists with family assessment and intervention training)	*	Portuguese	Yes	No	None
Traumatic Events Questionnaire (TEQ)	from developers	free	11+	82.5	Self-completion paper-pencil	5-10 minutes	English, Hebrew	Yes	No	None
Traumatic Event Screening Instrument for Children (TESI-C)	National Centre for PTSD, Dartmouth	*	12+	76.3	Interview by clinician	45-90 minutes	English	Yes	No	clinician interviewers
Things I have seen and heard (TISH)	Open access (citation required)	Free	NA	88.1	Interviewer administered	5-10 minutes	English	Yes	No	None
**Only sexual violence**										
Checklist of sexual abuse and related events (young child) (C-SARS)	Open access (citation required)	Free	Interviewer applied age 5-12	Interview (no reading)	Interviewer led	*	English	*	No	*
Child Sexual Abuse Questionnaire (CSAQ1)	Part of paper	Free	12	73	Self-completion	*	German, French and Italian	*	*	*
Childhood Sexual Abuse Questionnaire (CSAQ2)	*	*	*	73	Self-completion	*	Hebrew	*	*	*

Sexual Abuse Exposure Questionnaire (SAEQ)	Request to use	*	16+	62	*	30-45 minutes	English	Yes	No	*
Sexual Experiences Survey (SES)	Open access (citation required)	Free	14+	61.8	Self-completion; paper-pencil	*	English	Yes	No	None
**Only psychological violence**										
Comprehensive Assessment of Psychological Maltreatment - Child Version (CAPM-CV)	*	*	10+	Not retrievable	Self-completion	*	English	*	*	*
**Only physical violence**										
Alabama Parenting Questionnaire (APQ)	Open access (citation required)	Free	6+	85.4	Self-completion with paper and pencil or telephone	*	English, German	Yes	No	None
Ghent Parental Behaviour Scale (GPBS)	*	*	7+	89.1	Self-completion with support by an interviewer	*	Dutch	*	*	*
Mexican Parenting Questionnaire Adolescent Version (MPQ)	Part of paper	*	11+	83.3	Self-report	*	Spanish, English	*	No	None
Multiple Indicator Cluster Survey (MICS)	Open access (citation required)	Free	NA	83.7	Multiple (interview and/or self-completion)	*	English; French; Spanish; Arabic; Russian (plus many other translations)	Yes	No	None
Scale of Parental Discipline (SPaD)	Open access (citation required)	Free	10+	93.9	Paper-pencil self-completion	*	English	*	No	*
Schedule for Risk Factors (SRF)	*	*	NA	Not retrievable	Interview	15 minutes	English, Spanish	*	No	*
Schedule for the Assessment of IPV Exposure in Children (SAIPVEC)	*	*	NA	available only in Spanish	Interview	*	Spanish	*	*	*
Violence History Questionnaire (VHQ)	*	*	Children	Not retrievable	Self-completion	*	English	Yes (Dalenberg 1983)	*	*
**Only Neglect**										
Child Neglect Questionnaire (CNQ)	Part of paper	Free	10+	92.2	Self-completion	20 minutes	English	No	No	None
Multidimensional	Open access	Free	Children	68.8	Interview and	30 minutes	English, Turkish	Yes	No	*

neglectful behavior scale (MNBS-CR)	(citation required)	questionnaire

* denotes that no information was available

Supplement 5: Methodological quality content validity studies

Assessment of methodological quality of development and content validity studies

		Development Study Quality			Content Validity Study Quality				
		Item generation	Cognitive Interview		Asking children			Asking Professionals	
**Instrument**	**Reference**	**Relevance**	**Comprehensiveness**	**Comprehensibility**	**Relevance**	**Comprehensiveness**	**Comprehensibility**	**Relevance**	**Comprehensiveness**
**Multiple forms of violence**									
CAS1	Malik & Shah, 2007	Doubtful	Doubtful	Doubtful	*	*	Doubtful	Doubtful	Doubtful
CEVQ	Walsh et al., 2008	*	*	*	Doubtful	Doubtful	Doubtful	Doubtful	Doubtful
CECA	Shirinbayan et al., 2020	*	*	Very good	*	*	Very good	*	*
CASRS	Mohammadkhani et al., 2003	Adequate	*	*	Adequate	*	*	Doubtful	*
CTScreen	Lang & Connell, 2017	Very good	Doubtful	Very good	*	*	*	*	*
ICAST-CH	Silveira et al., 2016	*	*	*	*	*	Doubtful	*	*
	Zolotor et al., 2009	Inadequate	*	*	*	*	*	*	*
	Sahaimi et al., 2020	*	Inadequate	Inadequate	*	*	*	*	*
ICAST-CI	Zolotor et al., 2009	Inadequate	*	*	*	*	*	*	*
ICAST-Trial C	Meinck et al., 2018	*	*	*	Very good	*	Very good	*	*
JVQ	Hamby et al., 2000	*	*	*	Adequate	Adequate	Adequate	Adequate	Adequate

* denotes could not be assessed due to lack of information

Supplement 6: Quality of content validity studies

Quality of content validity for each included study on the development and content validity of an instrument

Instrument	Reference	Relevance			Comprehensiveness			Comprehensibility			
		Development Study	Content Validity	Reviewer Rating	Development Study	Content Validity	Reviewer Rating	Development Study	Content Validity	Reviewer Rating	Language
					**Multiple forms of violence**						
CAS1	Malik & Shah, 2007	+	?	+	?	+	+	?	?	+	Urdu/English
CEVQ	Walsh et al., 2008	*	±	+	*	+	+	*	?	+	English
CECA	Shirinbayan et al., 2020	*	*	+	*	*	+	*	±	+	Persian
CASRS	Mohammadkhani et al. 2003	+	?	+	*	*	?	*	*	+	Persian, English
CTScreen	Lang & Connell 2017	+	*	+	?	*	?	+	*	+	English
ICAST-CH	Silveira et al., 2016	*	*	*	*	*	*	*	+	?	Portuguese
	Zolotor et al., 2009	±	*	+	*	*	+	*	*	+	English, Arabic, Turkish, Portuguese
	Sahaimi et al., 2020	±	*	?	-	*	?	-	*	?	Malay
ICAST-CI	Zolotor et al., 2009	±	?	+	*	*	+	*	*	+	English, Arabic, Turkish, Portuguese
ICAST-Trial C	Meinck et al., 2018	?	+	+	?	-	+	?	±	+	Xhosa
JVQ	Hamby et al., 2000	*	+	+	*	+	+	*	+	+	English

Note. +sufficient rating, ? indeterminate rating, -insufficient rating, ± inconsistent rating, * denotes could not be assessed due to lack of information

Supplement 7: Overall content validity quality rating

Methodological and content validity quality assessment of development and content validity studies per instrument

Instrument	Relevance		Comprehensiveness		Comprehensibility	
	**Overall quality of content validity (see T4)**	**Quality of evidence**	**Overall quality of content validity**	**Quality of evidence**	**Overall quality of content validity**	**Quality of evidence**
			**Multiple forms of violence**			
CAS1	±	Low	+	Moderate	+	Moderate
CEVQ	+	Low	+	Moderate	±	Moderate
CECA	+	Very good	+	Moderate	+	High
CASRS	+	Moderate	NE	NE	NE	NE
CTScreen	+	High	*	Low	+	High
ICAST-CH	±	Very low	±	Very low	±	Low
ICAST-CI	±	Very low	*	*	*	*
ICAST-Trial C	±	High	±	High	+	High
JVQ	+	Moderate	+	Moderate	+	Moderate

Note. Quality rating based on GRADE criteria: high level of confidence, moderate level of confidence, low level of confidence, very low level of confidence, Not evaluated (NE – instrument could not be retrieved)
+sufficient rating, ? indeterminate rating, -insufficient rating, ± inconsistent rating, * denotes could not be assessed due to lack of information

Supplement 8: Methodological quality of psychometric studies

Methodological Quality Assessment of Psychometric studies

Instrument	Reference	Structural validity	Internal consistency	Cross-cultural validity	Reliability	Measurement error	Criterion Validity	Hypothesis testing	Responsiveness	Concordance	Language
					**Multiple forms of violence**						
ACE-ASF	Meinck et al., 2017	Very good	Very good	Inadequate	*	*	*	Very good	*	*	Romanian
ACE-IQ	Kidman et al., 2019	Very good	*	*	*	*	*	Very good	*	*	chiChichewa
ACE	Matsuura et al., 2009	*	*	*	*	*	*	Inadequate	*	*	Japanese
M-ACE	Finkelhor et al., 2015	*	*	*	*	*	*	Doubtful	*	*	English
CAS1	Malik & Shah, 2007	Inadequate	Doubtful	*	*	*	*	*	*	*	Urdu/English
CASRS	Baniasad et al., 2016	*	Inadequate	*	*	*	*	Doubtful	*	*	Farsi
	Mohammadkhani et al., 2003	Very good	Very good	*	Very good	*	Adequate	Very good	*	*	Persian, English
CAS2	Esposito et al 2002	*	*	*	*	*	*	Very good	*	*	English
CMIS-SF	Munoz 1996	*	*	*	*	*	*	Inadequate	*	*	English
CMQ	Sesar et al., 2008	*	*	*	*	*	*	Adequate	*	*	Croatian
CTS	Lang et al., 2017	*	*	*	Adequate	*	*	Very good	*	Adequate	English
	Lang et al., 2018	*	Very good	*	Very good	*	*	Very good	*	Adequate	English
CECA	Shirinbayan et al., 2020	Very Good	Adequate	*	Very Good	*	*	*	*	*	Persian
CEVQ	Walsh et al 2008	*	*	*	Adequate	*	*	Doubtful	*	Adequate	English
	Tanaka et al 2012	*	Very good	*	Doubtful	*	*	Very good	*	Adequate	English
	Fuller-Thomson et al 2016	*	*	*	*	*	*	Very good	*	*	English
	Tardif-Williams et al 2017	*	Very good	*	*	*	*	Very good	*	*	English

CTQ-SF	Mikaeili et al 2013[2]	*	*	*	*	*	*	Doubtful	*	*	Farsi
	Zimmerman 2006	*	Very good	*	*	*	*	Very good	*	*	Farsi
	Tanaka et al 2011	*	Doubtful	*	*	*	*	Doubtful	*	*	English
	Wekerle et al 2001	*	Inadequate	*	*	*	*	Adequate	*	Adequate	English
	Crooks et al 2011	*	*	*	*	*	*	very good	*	*	English
	Smith 2006	*	*	*	*	*	*	Doubtful	*	*	English
	Leguizamo 2000	*	*	*	*	*	*	Doubtful	*	Doubtful	English
	Zhang et al 2017	*	Very good	*	*	*	*	Very good	*	*	English
	Peh et al 2017	*	Inadequate	*	*	*	*	Adequate	*	*	English
	Wolfe et al 2001	*	*	*	Inadequate	*	*	Very good	*	*	English
	van Vugt et al 2014	*	Doubtful	*	*	*	*	Doubtful	*	*	French
	Maciel et al 2013	*	*	*	*	*	*	Adequate	*	*	Portuguese
	Horesh et al 2008	*	Inadequate	*	*	*	*	Very good	*	*	Portuguese
	Shamu et al 2016	*	*	*	*	*	*	Doubtful	*	*	English, SeTswana, SePedi
	Chung et al 2017	*	*	*	*	*	*	Doubtful	*	*	Chinese
	Lupis 2015	*	*	*	*	*	*	Adequate	*	*	Croatian
	Boonmann et al 2016	*	*	*	*	*	*	Adequate	*	*	Dutch
	Krischer et al 2008	*	Very good	*	*	*	*	Very good	*	*	German
	Ometto et al 2016	*	*	*	*	*	*	Very good	*	*	Portuguese
	Dias et al 2015	*	*	*	*	*	*	Very good	*	*	Portuguese
	Miller 2011	*	Very good	*	*	*	*	*	*	*	English
	Stoltz 2007	*	Doubtful	*	*	*	*	*	*	*	English
	Burton 2008	*	Very good	*	*	*	*	*	*	*	English
	Charak 2016	Very good	Very good	*	*	*	*	Doubtful	*	*	Kirundi
	Mizuki 2020	*	Very good	*	*	*	*	*	*	Very good	Japanese
CTQ	Levendosky 2002	*	*	*	*	*	*	Doubtful	*	*	English
	Bernstein 1997	Adequate	Very good	*	*	*	*	*	*	Very good	English

	Lipschitz 1999	*	*	*	*	*	*	*	*	Adequate	English
	Brockie 2015	*	Very good	*	*	*	*	Adequate	*	*	English
	Ungar 2004	*	*	*	*	*	*	Doubtful	*	*	English
CMH-SR	Bhat et al 2012	*	*	*	*	*	*	Adequate	*	*	English/Hindi
CARI	Crowley et al 2003	*	*	*	*	*	*	Very good	*	*	English
CTI2	Barnes et al 2009	*	*	*	Doubtful	*	*	Doubtful	*	*	English
	Negriff et al 2017	*	*	*	*	*	*	Very good	*	*	English
ETISR-SF	Park et al 2018	*	Inadequate	*	Doubtful	*	*	Adequate	*	*	Korean
FAST	Cecil et al 2016	*	Inadequate	*	*	*	*	Very good	*	*	English
ICAST-CH	Zolotor et al 2009	*	Very good	*	*	*	*	*	*	*	English, Arabic, Turkish, Portuguese
	Sofuoglu et al 2016	*	*	*	*	*	*	*	*	Inadequate	Turkish
	Hosseinkhani et al 2015	*	Inadequate	*	*	*	*	*	*	*	Farsi
	Usta et al 2013	*	Very good	*	*	*	*	Very good	*	*	Arabic
	Chang et al 2013	Very good	Very good	*	*	*	*	*	*	*	Chinese
	Ajdukovic et al 2013[1]	*	Very good	*	*	*	*	*	*	*	Croatian
	Meinck et al 2020	Very good	Very good	Very good	*	*	*	*	*	*	Greek, Turkish, Bulgarian, Serbian, Croatian, Macedonian, Bosnian, Romanian, Albanian
	Sahaimi et al 2020	Adequate	Very good	*	*	*	*	*	*	*	Malay
ICAST-CI	Zolotor et al 2009	*	Very good	*	*	*	*	*	*	*	
	Devries et al 2017	*	*	*	*	*	*	Inadequate	*	*	Luganda
ICAST-Trial C	Meinck et al 2018	Very good	Very good	*	*	*	*	Very good	Very good	*	isiXhosa
SC-ICAST CH	Feng et al 2020	Very good	Very good	*	*	*	Very good	*	*	*	Chinese
JVQ	Turner 2011	*	*	*	*	*	*	Very good	*	*	English

	Turner 2010	*	*	*	*	*	*	Very good	*	*	English
	Finkelhor 2005a	*	*	*	*	*	*	Very good	*	*	English
	Cuevas 2009	*	*	*	*	*	*	Very good	*	*	English
	Soler 2013	*	Inadequate	*	*	*	*	Doubtful	*	*	Spanish
	Finkelhor 2005b	*	Inadequate	*	Doubtful	*	*	Very good	*	*	English
JVQ (15 item version)	Pitcairn 2006	*	*	*	*	*	*	Very good	*	*	English
	Chan 2011	*	Very good	*	*	*	*	Very good	*	*	Chinese
	Pereda 2016	*	*	*	*	*	*	Very good	*	*	Spanish
	Bashir 2015	*	Very good	*	*	*	*	Doubtful	*	*	Urdu
	Forns 2013	Very good	Very good	*	*	*	*	Adequate	*	Adequate	Spanish, Catalan
	Finkelhor 2013	*	*	*	*	*	*	Very good	*	*	English
KID-SAVE	Hilker 2002	*	*	*	*	*	*	Very good	*	*	English
	Flowers 2000	Doubtful	Very good	*	Doubtful	*	*	Very good	*	*	English
LITE	Nilsson et al 2010	*	*	*	very good	*	*	Very good	*	*	Swedish
	Tingskull et al 2015	*	*	*	Adequate	*	*	*	*	*	Swedish
	Greenwald et al 1999	*	*	*	*	*	*	Very good	*	*	English
LONGSCAN	Nooner 2007	*	*	*	*	*	*	*	*	Very good	English
	Everson et al. 2008	*	*	*	*	*	*	*	*	Very good	English
pediMACE	Isele 2016	very good	*	*	*	*	*	Inadequate	*	*	Swahili
	Hecker et al 2016[3]	*	*	*	*	*	*	inadequate	*	*	swahili
MACE	Seiler et al 2016	*	Very good	*	*	*	*	Doubtful	*	*	Spanish
CTS-PC-P	Sierau et al 2018	very good	Very good	*	*	*	*	Very good	*	very good	German
CTS-PC	Edwards 2014	*	Very good	*	*	*	*	Very good	*	*	English
	Shaffer et al 2012	*	Very good	*	*	*	*	*	Very good	*	English
	Kobulsky et al 2017	*	*	*	Very good	*	*	Very good	*	*	English

	Kobulsky et al 2016	*	*	*	*	*	*	Very good	*	*	English
	Schaeffer et al 2013	*	*	*	*	*	*	*	Inadequate	*	English
	Lindhiem et al 2014	*	*	*	Inadequate	*	*	*	Very good	*	English
	Kolko 1996	*	Doubtful	*	*	*	*	Very good	*	*	English
	Schneider et al 2015	*	*	*	Inadequate	*	*	*	*	*	English
	Donovan 2009	*	Very good	*	*	*	*	Doubtful	*	*	English
	De Zoysa et al 2006	*	Very good	*	Doubtful	*	*	Inadequate	*	*	Sinhalese
PPS	Hernandez-Guzman et al. 2013	Very good	Very good	*	*	*	*	Very good	*	*	Spanish
PPI2	Cole et al. 2014	*	Very good	*	*	*	*	*	*	*	English
	Tucker et al. 2014	*	Inadequate	*	*	*	*	*	*	*	English
	Salamone 2006	*	*	*	Adequate	*	*	*	Adequate	*	English
SIFAR	Dos Santos et al. 2016	Doubtful	Very good	*	*	*	*	*	*	*	Portuguese
TEQ	Lev-Wiesel et al. 2014	*	*	*	*	*	*	Adequate	*	*	Hebrew
TESI	Tangeman 2004	*	*	*	*	*	*	Adequate	*	*	English
TISH	Nordstrom-Klee 2001	*	Doubtful	*	*	*	*	Doubtful	*	*	English
	Richters and Martinez 1993	*	*	*	Doubtful	*	*	*	*	*	English
VHQ	Dingwall 1997	*	*	*	*	*	*	Doubtful	*	*	English
**Only sexual violence**											
C-SARS	O'Boyle 2002	*	*	*	*	*	*	Very good	*	*	English
CSAQ2	Horesh et al 2009	*	*	*	*	*	*	Inadequate	*	*	Hebrew
CSAQ1	Mohler Kuo et al 2014	*	Very good	*	*	*	*	*	*	*	German, Italian, French
SAEQ	Ungar 2004	*	*	*	*	*	*	Adequate	*	*	English

	Gillick 1997	*	*	*	*	*	*	Adequate	*	*	English
SES	Gidycz and Koss 1989	*	*	*	*	*	*	Very good	*	Adequate	English
**Only psychological violence**											
CAPM	Melmed 2012	*	Very good	*	*	*	*	Very good	*	*	English
	Edwards 2014	*	Very good	*	*	*	*	Very good	*	*	English
**Only physical violence**											
APQ	Zlomke et al 2015	Adequate	Very good	*	*	*	*	*	*	*	English
	Essau et al 2006	Very good	Very good	Doubtful	*	*	*	Very good	*	*	German
	Kjellgren et al 2013	*	Inadequate	*	*	*	*	*	Adequate	*	Swedish
GPBS	Van Leeuwen et al 2004	very good	Very good	*	Doubtful	*	*	Very good	*	*	Dutch
MICS	Betancourt et al. 2014	*	Very good	*	*	*	*	*	Adequate	*	English and Kinyarwanda
	Puffer et al. 2017	*	*	*	*	*	*	*		*	Karen and Burmese
MPQ	Halgunseth et al 2017	Very good	Very good	*	*	*	*	Very good	*	*	Spanish
SAIPVEC	de la Vega et al. 2011	*	*	*	*	*	*	Very good	*	*	Spanish
SPaD	Skillings 2006	Adequate	Doubtful	*	Very good	*	*	Adequate	*	*	English
SRF	Penelo et al. 2012	*	Very good	*	*	*	*	Adequate	*	*	Spanish
**Neglect**											
CNQ	Stewart et al., 2015	Inadequate	Very good	*	Doubtful	*	*	Adequate	*	*	English
MNBS-CR	Dubowitz et al. 2011	Adequate	*	*	*	*	*	Adequate	*	*	English
	Kantor et al. 2004	*	*	*	Very good	*	*	Very good	*	Very good	English
	Beyazit and Ayhan 2019	Inadequate	Very good	*	*	*	*	Very good	*	*	Turkish

Note. Quality rating based on GRADE criteria: high level of confidence, moderate level of confidence, low level of confidence, very low level of confidence, Not evaluated (NE – instrument could not be retrieved), * denotes could not be assessed due to lack of information

[1]only used sexual abuse scale
[2] did not use sexual abuse scale
[3]only uses harsh discipline subscal
+sufficient rating, ? indeterminate rating, -insufficient rating, ± inconsistent rating

Supplement 9: Quality of psychometric properties

Quality of the psychometric properties of each included study

Instrument	Reference	Structural validity	Internal consistency	Cross-cultural validity	Reliability	Measurement error	Criterion Validity	Hypothesis testing	Responsiveness	Concordance
				**Multiple forms of violence**						
ACE-ASF	Meinck et al., 2017	+	±	+	*	*	*	+	*	*
ACE-IQ	Kidman et al., 2019	+	*	*	*	*	*	±	*	*
ACE	Matsuura et al., 2009	*	*	*	*	*	*	+	*	*
M-ACE	Finkelhor et al., 2015	*	*	*	*	*	*	+	*	*
CAS1	Malik & Shah, 2007	?	-	*	*	*	*	*	*	*
CASRS	Baniasad et al., 2016	*	?	*	*	*	*	+	*	*
	Mohammadkhani et al., 2003	+	+	*	+	*	?	+	*	*
CAS2	Esposito et al 2002	*	*	*	*	*	*	+	*	*
CMIS-SF	Munoz 1996	*	*	*	*	*	*	-	*	*
CMQ	Sesar et al., 2008	*	*	*	*	*	*	+	*	*
CTS	Lang and Connell 2017	*	*	*	-	*	*	+	*	+
	Lang and Connell 2018	*	+	*	+	*	*	+	*	?
CECA	Shirinbayan et al., 2020	+	±	*	+	*	*	*	*	*
CEVQ	Walsh et al 2008	*	*	*	+	*	*	+	*	+
	Tanaka et al 2012	*	+	*	+	*	*	+	*	-
	Fuller-Thomson et al 2016	*	*	*	*	*	*	+	*	*
	Tardif-Williams et al 2017	*	+	*	*	*	*	+	*	*
CTQ-SF	Mikaeili et al 2013[2]	*	*	*	*	*	*	+	*	*
	Zimmerman 2007	*	+	*	*	*	*	+	*	*
	Tanaka et al 2011	*	±	*	*	*	*	±	*	*
	Wekerle et al 2001	*	?	*	*	*	*	+	*	+

	Crooks et al 2011	*	*	*	*	*	*	+	*	*
	Smith 2006	*	*	*	*	*	*	+	*	*
	Leguizamo 2000	*	*	*	*	*	*	+	*	±
	Zhang et al 2017	*	±	*	*	*	*	+	*	*
	Peh et al 2017	*	?	*	*	*	*	+	*	*
	Wolfe et al 2011	*	*	*	?	*	*	+	*	*
	van Vugt et al 2014	*	+	*	*	*	*	+	*	*
	Maciel et al 2013	*	*	*	*	*	*	+	*	*
	Horesh et al 2008	*	?	*	*	*	*	+	*	*
	Horesh et al 2009	*	*	*	*	*	*	+	*	*
	Shamu et al 2017	*	*	*	*	*	*	+	*	*
	Chung et al 2017	*	*	*	*	*	*	+	*	*
	Lupis 2015	*	*	*	*	*	*	+	*	*
	Boonmann et al 2016	*	*	*	*	*	*	±	*	*
	Krischer et al 2008	*	+	*	*	*	*	+	*	*
	Ometto et al 2016	*	*	*	*	*	*	+	*	*
	Dias et al 2015	*	*	*	*	*	*	+	*	*
	Miller 2011	*	+	*	*	*	*	*	*	*
	Stoltz 2007	*	+	*	*	*	*	*	*	*
	Burton 2008	*	+	*	*	*	*	*	*	*
	Charak 2017	-	-[a]	*	*	*	*	+	*	*
	Mizuki 2020	*	+	*	*	*	*	*	*	+
CTQ	Levendosky 2002	*	*	*	*	*	*	+	*	*
	Bernstein 1997	?	+	*	*	*	*	*	*	+
	Lipschitz 1999	*	*	*	*	*	*	*	*	+
	Brockie 2015	*	-[a]	*	*	*	*	+	*	*
	Ungar 2004	*	*	*	*	*	*	+	*	*
CMH-SR	Bhat et al. 2012	*	*	*	*	*	*	+	*	*

CARI	Crowley et al 2003	*	*	*	*	*	*	+	*	*
CTI2	Barnes et al 2009	*	*	*	±	*	*	+	*	*
	Negriff et al. 2017	*	*	*	*	*	*	+	*	*
ETISR-SF	Park et al. 2017	*	+	*	±	*	*	±	*	*
FAST	Cecil et al. 2017	*	+	*	*	*	*	±	*	*
ICAST-CH	Zolotor et al. 2009	*	-	*	*	*	*	*	*	*
	Sofuoglu et al. 2016	*	*	*	*	*	*	*	*	-[b]
	Hosseinkhani et al. 2016[2]	*	+	*	*	*	*	*	*	*
	Usta et al. 2013[2]	*	+	*	*	*	*	+	*	*
	Chang et al. 2013	-	-	*	*	*	*	*	*	*
	Adjukovic et al. 2013	*	-	*	*	*	*	*	*	*
	Meinck et al. 2020	+	-	+	*	*	*	*	*	*
	Sahaimi et al. 2020	-	-	*	*	*	*	*	*	*
ICAST-CI	Zolotor et al. 2009	*	+	*	*	*	*	*	*	*
	Devries et al. 2017	*	*	*	*	*	*	+	*	*
ICAST-Trial C	Meinck et al. 2018	+	-	*	*	*	*	+	+	*
SC-ICAST-CH	Feng et al. 2020	-	-	*	*	*	+	*	*	*
JVQ	Turner 2010	*	*	*	*	*	*	+	*	*
	Finkelhor 2005a	*	*	*	*	*	*	+	*	*
	Cuevas 2009	*	*	*	*	*	*	+	*	*
	Soler 2013	*	?	*	*	*	*	+	*	*
	Finkelhor 2005b	*	-	*	-	*	*	+	*	*
	Pitcairn 2006	*	*	*	*	*	*	+	*	*
	Chan 2011	*	+	*	*	*	*	+	*	*
	Pereda 2016	*	*	*	*	*	*	+	*	*
	Bashir 2015	*	-	*	*	*	*	+	*	*
	Forns 2013	+	-	*	*	*	*	+	*	+
	Finkelhor 2013	*	*	*	*	*	*	+	*	*

KID-SAVE	Hilker 2002	*	*	*	*	*	*	+	*	*
	Flowers 2000	?	±	*	±	*	*	+	*	*
LITE	Nilsson et al. 2010	*	*	*	-	*	*	+	*	*
	Tingskull et al. 2015	*	*	*	-	*	*	*	*	*
	Greenwald et al. 1999	*	*	*	*	*	*	+	*	*
LONGSCAN	Nooner 2007	*	*	*	*	*	*	*	*	-
	Everson et al. 2008	*	*	*	*	*	*	*	*	-
pediMACE	Isele 2016	-	*	*	*	*	*	+	*	*
	Hecker et al. 2016[3]	*	*	*	*	*	*	+	*	*
MACE	Seiler et al. 2016	*	+	*	*	*	*	±	*	*
CTS-PC-P	Sierau et al. 2018	-	+	*	*	*	*	+	*	-
CTS-PC	Edwards 2014	*	±	*	*	*	*	±	*	*
	Shaffer et al. 2012	*	-	*	*	*	*	*	+	*
	Kobulsky et al. 2017	*	*	*	-	*	*	+	*	*
	Kobulsky et al. 2016	*	*	*	*	*	*	+	*	*
	Schaeffer et al. 2013	*	*	*	*	*	*	*	-	*
	Lindhiem et al. 2014	*	*	*	-	*	*	*	+	*
	Kolko 1996	*	+	*	*	*	*	+	*	*
	Schneider et al. 2015	*	*	*	-	*	*	*	*	*
	Donovan 2009	*	+	*	*	*	*	+	*	*
	De Zoysa et al. 2006	*	±	*	±	*	*	+	*	*
PPS	Hernandez-Guzman et al. 2013	+	+	*	*	*	*	+	*	*
PPI2	Cole et al. 2014	?	+	*	*	*	*	*	*	*
	Tucker and Rodriguez 2014	-	-	*	*	*	*	*	*	*
	Salamone 2006	*	*	*	+	*	*	*	-	*
SIFAR	Dos Santos and Alberto 2016	?	-	*	*	*	*	*	*	*
TEQ	Lev-Wiesel and Zohar 2014	*	*	*	*	*	*	+	*	*
TESI	Tangeman 2004	*	*	*	*	*	*	+	*	*

TISH	Nordstrom-Klee 2001	*	-	*	*	*	*	+	*	*
	Richters and Martinez 1993	*	*	*	+	*	*	*	*	*
VHQ	Dingwall 1997	*	*	*	*	*	*	+	*	*
**Only sexual violence**										
C-SARS	O-Boyle, 2002	*	*	*	*	*	*	-	*	*
CSAQ1	Mohler-Kuo et al. 2014	*	-	*	*	*	*	*	*	*
CSAQ2	Horesh et al 2009	*	*	*	*	*	*	+	*	*
SAEQ	Ungar 2004	*	*	*	*	*	*	+	*	*
	Gillick 1996	*	*	*	*	*	*	+	*	*
SES	Gidycz and Koss 1989	*	*	*	*	*	*	+	*	+
**Only psychological violence**										
CAPM	Melmed 2012	*	+	*	*	*	*	+	*	*
	Edwards 2014	*	+	*	*	*	*	+	*	*
**Only physical violence**										
APQ	Zlomke et al 2015	-	+	*	*	*	*	*	*	*
	Essau et al 2006	-	+	-	*	*	*	+	*	*
	Kjellgren et al 2013	*	+	*	*	*	*	*	+	*
GPBS	Van Leeuwen et al. 2004	-	-	*	-	*	*	+	*	*
MICS	Betancourt et al. 2014	*	-	*	*	*	*	*	+	*
	Puffer et al. 2017	*	*	*	*	*	*	*	+	*
MPQ	Halgunseth et al. 2017	+	-	*	*	*	*	+	*	*
SPaD	Skillings 2006	+	-	*	+	*	*	+	*	*
SRF	Penelo et al. 2012	*	-	*	*	*	*	+	*	*
SAIPVEC	de la Vega et al. 2011	*	*	*	*	*	*	+	*	*
**Neglect**										
CNQ	Stewart et al. 2015	?	+	*	-	*	*	+	*	*
MNBS-CR	Beyazit and Ayhan 2019	+	-	*	*	*	*	+	*	*
	Dubowitz et al. 2011	+	*	*	*	*	*	±	*	*

Kantor et al. 2004	*	*	*	+	*	*	+	*	+

Note. [1]only used sexual abuse scale

[2] did not use sexual abuse scale

[3]only uses harsh discipline subscal

+sufficient rating, ? indeterminate rating, -insufficient rating, ± inconsistent rating, * denotes could not be assessed due to lack of information

Supplement 10: Overall quality rating of psychometric properties

Overall quality of the psychometric properties and evidence quality per instrument

Instrument	Structural validity		Internal consistency		Cross-cultural validity		Reliability		Measurement error		Criterion Validity		Hypothesis testing		Responsiveness		Concordance	
	Overall rating	Quality of Evidence	Overall rating	Quality of Evidence	Overall rating	Quality of Evidence	Overall rating	Quality of Evidence	Overall rating	Quality of Evidence	Overall rating	Quality of Evidence	Overall rating	Quality off Evidence	Overall rating	Quality of Evidence	Overall rating	Quality of Evidence
**Multiple forms of violence**																		
ACE	*	*	*	*	*	*	*	*	*	*	*	*	+	Very low	*	*	*	*
ACE-ASF	+	High	±	High	+	Very low	*	*	*	*	*	*	*	High	*	*	*	*
ACE-IQ	+	High	*	*	*	*	*	*	*	*	*	*	±	High	*	*	*	*
CARI	*	*	*	*	*	*	*	*	*	*	*	*	+	High	*	*	*	*
CAS1	?	Very low	-	Very low	*	*	*	*	*	*	*	*	*	*	*	*	*	*
CAS2	*	*	*	*	*	*	*	*	*	*	*	*	+	High	*	*	*	*
CASRS	+	High	+	Moderate	*	*	+	High	*	*	?	Moderate	+	Moderate	*	*	*	*
CECA	+	High	±	Moderate	*	*	+	High	*	*	*	*	*	*	*	*	*	*
CEVQ	*	*	+	High	*	*	+	Moderate	*	*	*	*	+	Moderate	*	*	±	Moderate
CMIS-SF	*	*	*	*	*	*	*	*	*	*	*	*	-	Low	*	*	*	*
CMQ	*	*	*	*	*	*	*	*	*	*	*	*	+	Moderate	*	*	*	*
CTI2	*	*	±	NE	*	*	*	*	*	*	*	*	+	High	*	*	*	*
CTQ	?	Moderate	±	High	*	*	*	*	*	*	*	*	+	Moderate	*	*	+	High
CTQ-SF	*	*	±	NE	*	*	?	Very low	*	*	*	*	+	Moderate	*	*	±	High
CMH-SR	*	*	*	*	*	*	*	*	*	*	*	*	+	Moderate	*	*	*	*
CTS-PC	*	*	±	NE	*	*	-	Moderate	*	*	*	*	+	High	±	NE	*	*
CTS-PC-P	-	High	+	High	*	*	*	*	*	*	*	*	+	High	*	*	-	High
CTS	*	*	+	High	*	*	±	High	*	*	*	*	+	High	*	*	?	High
ETISR-SF	*	*	+	Very low	*	*	±	NE	*	*	*	*	±	NE	*	*	*	*
FAST	*	*	+	Very low	*	*	*	*	*	*	*	*	±	NE	*	*	*	*
ICAST-CH	±	NE	±	NE	+	High	*	*	*	*	*	*	+	High	*	*	-	Low

ICAST-CI	*	*	+	Moderate	*	*	*	*	*	*	*	*	+	High	*	*	*	*
ICAST-Trial C	+	High	-	High	*	*	*	*	*	*	*	*	+	High	+	High	*	*
JVQ	+	High	-	Low	*	*	-	Low	*	*	*	*	+	High	*	*	+	High
KID-SAVE	?	Low	±	NE	*	*	±	NE	*	*	*	*	+	High	*	*	*	*
LITE	*	*	-	High	*	*	*	*	*	*	*	*	+	High	*	*	*	*
LONGSCAN	*	*	*	*	*	*	*	*	*	*	*	*	*	*	*	*	-	High
M-ACE	*	*	*	*	*	*	*	*	*	*	*	*	+	Low	*	*	*	*
MACE	*	*	+	High	*	*	*	*	*	*	*	*	±	NE	*	*	*	*
pediMACE	-	High	*	*	*	*	*	*	*	*	*	*	+	Very low	*	*	*	*
PPI2	*	*	-	Moderate	*	*	+	Moderate	*	*	*	*	*	*	-	Moderate	*	*
PPS	+	High	+	High	*	*	*	*	*	*	*	*	+	High	*	*	*	*
SC-ICAST-CH	-	High	-	High	*	*	*	*	*	*	+	High	*	*	*	*	*	*
SIFAR	?	Low	-	High	*	*	*	*	*	*	*	*	*	*	*	*	*	*
TEQ	*	*	*	*	*	*	*	*	*	*	*	*	+	Moderate	*	*	*	*
TESI	*	*	*	*	*	*	*	*	*	*	*	*	+	Moderate	*	*	*	*
TISH	*	*	-	Low	*	*	+	Low	*	*	*	*	+	Low	*	*	*	*
VHQ	*	*	*	*	*	*	*	*	*	*	*	*	+	Low	*	*	*	*
**Only sexual violence**																		
C-SARS	*	*	*	*	*	*	*	*	*	*	*	*	-	High	*	*	*	*
CSAQ1	*	*	-	High	*	*	*	*	*	*	*	*	*	*	*	*	*	*
CSAQ2	*	*	*	*	*	*	*	*	*	*	*	*	+	Low	*	*	*	*
SAEQ	*	*	*	*	*	*	*	*	*	*	*	*	+	Moderate	*	*	*	*
SES	*	*	*	*	*	*	*	*	*	*	*	*	+	High	*	*	+	Moderate
**Only psychological violence**																		
CAPM	*	*	+	High	*	*	*	*	*	*	*	*	+	High	*	*	*	*
**Only physical violence**																		
APQ	-	High	+	High	-	Moderate	*	*	*	*	*	*	+	High	+	Moderate	*	*
GPBS	-	High	-	High	*	*	-	Low	*	*	*	*	+	High	*	*	*	*
MICS	*	*	-	High	*	*	*	*	*	*	*	*	*	*	+	Moderate	*	*

MPQ	+	High	-	High	*	*	*	*	*	*	*	*	+	High	*	*	*	*
SAIPVEC	*	*	*	*	*	*	*	*	*	*	*	*	+	High	*	*	*	*
SPaD	+	Moderate	-	Low	*	*	+	High	*	*	*	*	+	Moderate	*	*	*	*
SRF	*	*	-	High	*	*	*	*	*	*	*	*	+	Moderate	*	*	*	*
**Only Neglect**																		
CNQ	?	Very low	+	High	*	*	-	High	*	*	*	*	+	High	*	*	*	*
MNBS-CR	+	Moderate	-	High	*	*	+	High	*	*	*	*	+	Moderate	*	*	+	High

Note. Quality rating based on GRADE criteria: High level of confidence, Moderate level of confidence, Low level of confidence, very low level of confidence, Not evaluated

+sufficient rating, ? indeterminate rating, -insufficient rating, ± inconsistent rating

NE=Not evaluated as if results are inconsistent, results should not be summarised, and evidence should not be graded, * denotes could not be assessed due to lack of information
